# Optimization of Conditions for Feather Waste Biodegradation by Geophilic *Trichophyton ajelloi* Fungal Strains towards Further Agricultural Use

**DOI:** 10.3390/ijerph191710858

**Published:** 2022-08-31

**Authors:** Michał Możejko, Justyna Bohacz

**Affiliations:** Department of Environmental Microbiology, Faculty of Agrobioengineering, University of Life Sciences in Lublin, Leszczyńskiego 7 Street, 20-069 Lublin, Poland

**Keywords:** fungal keratinolysis, optimization, *Trichophyton ajelloi*, mineral forms of N and S, enzyme activity, chicken feather, keratin

## Abstract

The aim of the study was to optimize culture conditions and medium composition to accelerate the biodegradation of chicken feather waste by keratinolytic soil strains of *Trichophyton ajelloi*, which are poorly known in this respect, as well as to propose hitherto unconsidered culture conditions for these fungi in order to obtain a biopreparation with a high fertilization value. Different pH of the medium, incubation temperatures, amounts of chicken feathers, additional carbon sources, and culture methods were tested. The process of optimizing keratin biodegradation was evaluated in terms of measuring the activity of keratinase, protease, disulfide reductase, concentration of released soluble proteins and peptides, total pool of amino acids, ammonium and sulfate ions, changes in medium pH, and feather weight loss. It was found that the studied fungal strains were capable of decomposing and mineralizing keratin from feather waste. Regarding the fertilizer value of the obtained hydrolysates, it was shown that the release of sulfate and ammonium ions was highest in a stationary culture containing 2% feathers with an initial pH of 4.5 and a temperature of 28 °C. Days 14–21 of the culture were indicated as the optimal culture time for these fungi to obtain biopreparations of high fertilizing value.

## 1. Introduction

Progressive population growth contributes to increased food demand of both plant and animal origin, but also to the generation of by-products. Many of these include valuable products with significant potential for reuse. The unmanaged masses of these wastes cause environmental pollution through the release of toxic compounds and development of pathogenic microorganisms. Byproducts from poultry production with high recycling potential include keratin waste, i.e., horns, hooves, hair, feathers, etc. [1]. According to statistical data [2,3] the consumption of poultry meat has been growing each year and is estimated to have increased by 6% globally in the last five years, and by 32% in Poland. The resulting keratin waste materials, including feathers, can be reused due to their high protein (90%), nitrogen (12–18%), and organic sulfur (2–5%) content. The current legal regulations in the field of waste management [4,5] oblige local governments and entrepreneurs to act to reduce waste generation or transfer waste materials to recycling institutions [6]. 

Based on the aforementioned Regulation of the European Parliament and the European Council (EC), feather wastes are classified as category 3 waste materials. These are wastes which pose low risk to human and animal health that can be reused for various purposes. Environmentally friendly technologies dominate among the proposals regarding the possibility of managing this waste, i.e., composting with lignocellulosic waste [6,7,8,9,10,11], microbial processing to obtain bioproducts with high fertilizer and feed potential [12,13,14], and obtaining keratinolytic enzymes for medical, pharmaceutical, industrial and bioenergy applications [15]. Implementing biological methods of keratin waste management is an alternative option to existing physical and chemical methods [1], which have an adverse impact on the environment and generate additional costs from an economic perspective. 

Limitations in the widespread use of microbial technologies for processing keratin waste are caused by its low biodegradability. Native keratins are hard-to-degrade proteins, available as a nutritional substrate only to a small number of microorganisms. Among the keratinolytic microorganisms capable of utilizing native keratin as the sole source of C and energy, bacteria of the genera *Bacillus*, *Sarcina*, *Streptomyces*, and fungi from the group of dermatophytes and *Chrysosporium* can be mentioned [12,13,14,16,17,18,19,20,21,22]. In the group of dermatophytes, in addition to pathogenic species, there are saprotrophic species called geophilic dermatophytes, which do not show affinity to higher organisms [23,24]. Saprotrophic keratinomycetes, in addition to cultivated soils [25,26], soils with a constant supply of keratinous matter [14,24], and composts [10], colonize feathers and nests of birds or mammalian hair [27], as well as bottom sediments, sewage, and municipal waste [21,28]. In cultivated soils and keratin-based composts, keratinolytic fungi are considered natural biofertilizers, as they release mineral forms of nitrogen and sulfur during the enzymatic biodegradation of native keratin (feathers, hair, other animal remains), which are assimilable by plants [10,12]. 

One of the more common soil keratinomycetes is *Trichophyton ajelloi*, which belongs to geophilic dermatophytes [24,25,26,29]. There is little data in the literature comprehensively describing keratin breakdown by these fungi [20]. For example, Kačinová, et al. [20] showed that *Trichophyton ajelloi* produced significant amounts of keratinolytic enzymes and their biosynthesis was stimulated by limiting the availability of carbon sources in the culture medium, while temperature activated keratinase secretion. According to Kumar and Kushwaha [21], *Trichophyton ajelloi*, in relation to other keratinophilic (=keratinolytic) fungi such as *Trichophyton mentagrophytes*, *Microsporum gypseum*, *Myceliophthora vellerea*, *Myceliophthora fergusii*, *Chrysosporium tropicum*, or *Chrysosporium indicum* was characterized by lower activity of keratinolytic enzymes. However, keratinase activity is not the only determinant of fungal keratinolytic potential. Korniłłowicz–Kowalska [30,31] has shown that there are numerous indicators reflecting the level of keratinolytic activity of fungi, which, in addition to proteolytic and keratinolytic activity, include the concentration of released soluble proteins and peptides, (N–NH_2_), N–NH_4_^+^, and S–SO_4_^2−^ amino groups, culture medium alkalization, and feather weight loss. Bohacz [12] identified the latter as the most reliable indicator of the biodegradation process of chicken feathers and described it as an ‘economic coefficient’. The process of S-keratin mineralization was also pointed out by Bohacz, et al. [14] as another important aspect of chicken feather biodegradation. The latter authors pointed out that in addition to nitrogen mineralization, organic sulfur mineralization was the determinant of fungal keratinolysis efficiency. With regard to the economic aspects of waste keratin biodegradation, the time of complete solubilization of this waste is crucial. As reported by Cai and Zheng [32], keratin biodegradation by bacteria and actinomycetes was significantly shorter in comparison to fungi. According to Korniłłowicz–Kowalska [30,31], the complete solubilization of native feather keratin by keratinolytic fungi could even last about two months. However, during the so-called rapid lysis phase, which lasted 14–21 days, the most active strains of keratinolytic fungi were able to break down 65–85% of these waste [30,31].

In view of the presented data and the lack of implemented solutions in the field of environmentally friendly methods of managing keratin waste and recycling of valuable organic matter, there is a need or even an obligation to seek new technologies or optimize already recognized methods of processing this waste. The possibility of obtaining fertilizer preparations can undoubtedly be counted among these solutions. In recent years, there has been increasing interest in developing methods to obtain various microbial biopreparations for their soil application and fertilization and protection of plants [33]. This is particularly important in connection with environmental protection efforts, but is also relevant to harnessing the potential of microorganisms as natural biofertilizers. Considering the alarming data related to climate change due to mismanagement of natural resources, the timing of sourcing innovative solutions for environmental protection is also becoming important.

The present work addresses contemporary demands for environmental protection. Its primary goal was to optimize culture conditions and substrate composition toward both accelerating the biodegradation of valuable chicken feather waste by keratinolytic strains of *Trichophyton ajelloi*—a soil fungus poorly known in this regard—but also to obtain a biopreparation of high fertilizer value. The activity of enzymes involved in the biodegradation process, feather weight loss and, in particular, the content of organic and mineral forms of nitrogen and sulfur were used as criteria in assessing the impact of individual optimized factors. 

## 2. Materials and Methods

### 2.1. Isolation of Trichophyton ajelloi Strains

Three strains of *Trichophyton ajelloi*, designated as III, XII, and XIV, were used to study the optimization of culture conditions and medium composition for geophilic keratinolytic fungi. They were selected from 37 strains of this species isolated from 4 different cultivated soils in southeastern Poland (Cambisol, Leptosol, and Chernozem). It was also the dominant species in these soils. All three strains were isolated by the keratin bait method from brown soil with a sandy loam grain size, classified according to the World Reference Base classification [34] as Cambisol. A detailed characterization of the mycobiome of these soils and the relationship between the occurrence of keratinolytic and non–keratinolytic fungi and the physical and chemical properties, in particular grain size, is presented in the study of Bohacz, et al. [26]. In turn, the characteristics of keratinolytic activity of the entire *Trichophyton ajelloi* collection is currently the subject of another study (unpublished data). The *Trichophyton ajelloi* strains selected for the study, based on their preliminarily identified ability to release mineral forms of N and S during biodegradation of feather waste keratin, were classified as highly keratinolytic. 

### 2.2. Trichophyton ajelloi Identification 

#### 2.2.1. Characteristics of Morphological Features

Species identification in pure fungal cultures was carried out on the basis of macro- and micromorphological characteristics on Petri dishes and agar slants, respectively, with Sabouraud medium with the following composition (g dm^−3^): glucose—40; peptone—10; agar—20, distilled water—1 dm^3^, and in microcultures, which was described in detail in the study of Bohacz, et al. [26]. Macroscopic identification concerned the colony morphology of these fungi and their ability to produce pigment. Microscopic identification using an Olympus BX-41 microscope (Olympus, Tokyo, Japan) equipped with a CVIII4 camera (Olympus, Tokyo, Japan) and Cell-A software (Soft Imaging System GmbH, version (v.) 1.20, Münster, Germany) included hyphae structure, the presence and structure of micro- and macroconidia (anamorph), as well as the presence or absence of fruiting bodies (teleomorph). The species classification was based on the systematic keys listed in Bohacz, et al. [26].

#### 2.2.2. Identification Using Molecular Methods 

In order to verify the determination on the basis of phenotypic characteristics of the three strains of *Trichophyton ajelloi*, identification by PCR and nucleotide sequencing was carried out at the Institute of Biochemistry and Biophysics (oligo.pl), Polish Academy of Sciences, Warsaw, Poland. The isolation of genetic material (DNA) was carried out from the fungal cultures in Petri dishes using the manual method described in the study of Liu and Paterson [35]. This was followed by PCR reaction and sequencing of the reaction product templates using specific primers: ITS1: ′5–TCCGTAGGTGAACCTGGCGG–3′ and ITS4: ′5–TCCTCCGCTTATTGATATGC–3′. PCR products were purified and sequencing was performed using the BigDye Terminator Mix v3.1 kit and an ABI3730xl genetic analyzer and the aforementioned primers. The resulting readings were assembled into appropriate contigs, thereby obtaining a consensus sequence. The results were compared with the UNITE database (https://unite.ut.ee/ accessed on 9 July 2021). The exact conditions of the PCR reaction and nucleotide sequencing are described in the study of Bohacz [12].

The resulting sequences are available in the GenBank database under accession numbers: *Trichophyton ajelloi* strain ON 461895 designated as III, *Trichophyton ajelloi* strain ON 468679 designated as XII, *Trichophyton ajelloi* strain ON 461993 designated as XIV.

### 2.3. Preliminary Evaluation of the Effect of Temperature on the Growth of Trichophyton ajelloi Fungi

The effect of different temperatures (20, 28, 37, and 50 °C) on the growth of the tested *Trichophyton ajelloi* fungal strains was evaluated by inoculating the mycelium in the center of a Petri dish with Sabouraud and MAE medium (g dm^−3^): malt extract—12.75, glycerin—2.35, dextrin—2.75, peptone—0.78, agar—15.00, and distilled water to a final volume of 1 dm^3^. After 7 and 14 days of fungal culture, colony size (ø in mm), pigment production capacity, and the color of the obverse and reverse of the colonies were determined. The cultures were incubated in ST 350/350 incubators (Pol–Eko, Wodzisław Śląski, Poland).

### 2.4. Preliminary Evaluation of Hydrolytic Abilities of Trichophyton ajelloi Strains

Testing the ability of the fungal strains to degrade protein, starch and fats was carried out in Petri dishes with an appropriate agar medium. To determine the ability to degrade protein, the fungi were inoculated on a medium with gelatin as the only source of C, N, and energy with the following composition (g dm^−3^): gelatin—6; glucose—0.05; peptone—0.1; NaCl—3; K_2_HPO_4_—1.5; KH_2_PO_4_—0.5; agar—20; broth—5 cm^3^; and distilled water to a final volume of 1 dm^3^. For the evaluation of amylolytic activity, the plates were prepared with a medium containing starch and sucrose as a source of C and energy with the following composition (g dm^−3^): NaNO_3_—3; K_2_HPO_4_—1; MgSO_4_—0.5; KCl—0.5; sucrose—10; starch—20; agar—20; FeSO_4_—trace amounts; and distilled water to a final volume of 1 dm^3^. Assessment of the lipolytic capacity of fungi was carried out on a medium with butyrate with the following composition (g dm^−3^) broth—3; peptone—5; agar—20; butyrate—10 cm^3^, and distilled water to a final volume of 1 dm^3^. The established cultures were incubated in an incubator at 28 °C for up to one week. After this time, the ability of fungi to degrade these compounds was analyzed. To demonstrate the ability to degrade starch, the cultures that grew on the medium with starch were poured over with Lugol’s iodine, for protein degradation with Frazier’s reagent (HgCl_2_—15 g; HCl—20 cm^3^, distilled water to a final volume of 100 cm^3^). The appearance of the clear zone around the fungus growth indicated the secretion of extracellular amylolytic or proteolytic enzymes by these fungi, respectively. The presence of a clear zone around the growth of these fungi on the medium with fat indicated the ability to decompose fats. Cultures for each strain were prepared in triplicate. 

The preliminary assessment of the ability to degrade protein, starch, and fat was presented in the form of the Enzymatic Index (IE) according to the equation described by de Lima, et al. [36].

$$\mathrm{IE}=\frac{\mathrm{size}\;\mathrm{of}\;\mathrm{clear}\;\mathrm{zone}\;\left(ø\mathrm{mm}\right)}{\mathrm{size}\;\mathrm{of}\;\mathrm{colony}\;\left(ø\mathrm{mm}\right)}$$

### 2.5. Optimization of Medium Composition and Culture Conditions of Trichophyton ajelloi Strains in the Process of Feather Keratin Biodegradation

In order to select optimal culture parameters for the biodegradation of feather waste keratin by *Trichophyton ajelloi*, different carbon and nitrogen sources, medium pH, incubation temperature, and culture aeration were tested. The activity of enzymes involved in the degradation of keratin protein, i.e., protease, keratinase, and disulfide reductase, concentration of keratinolysis products, which included soluble proteins and peptides, amino and thiol groups, ammonium, and sulfate ions, determined in culture fluids, and feather weight loss measured after 42 days of culture, were used as criteria of the effect of individual factors. Clear culture fluid for the determination of the aforementioned parameters was separated from mycelial residues by centrifugation at 7000 rpm and 4 °C (Eppendorf Centrifuge 5430 R, Hamburg, Germany). 

#### 2.5.1. Fungal Culture

*Trichophyton ajelloi* cultures were carried out in 300 cm^3^ Erlenmeyer flasks containing 100 cm^3^ of liquid sterile basal mineral medium with the following composition (g dm^−3^): KH_2_PO_4_—1.5; NaCl—0.01; MgCl_2_ 6H_2_O—0.05, distilled water to a final volume of 1 dm^3^, pH = 4.5, 1% chicken feather. The basal medium composition (control) was based on data developed for keratinolytic fungi of the *Chrysosporium* group, except for pH [12]. Chicken feather waste was derived from the “Superdrób” Poultry Plant in Lublin, Poland. Chemical composition of chicken feather dry weight (%): N–total—12.83; N–overall—15.17; P—0.10; K—0.10; Ca—0.18; Mg—0.02; organic matter—98.78, and (mg kg^−1^): S–SO_4_—0; S–total—8693; N–NO_3_—26; N–NH_4_—137 [10]. Preparation of feathers for the experiment and their sterilization, without disturbing the keratin structure, were carried out according to the procedure described by Bohacz, et al. [13]. Feathers were thoroughly rinsed (washed in a poultry slaughterhouse) with running and distilled water. After drying at 55 °C, they were cut into < 1.0 cm sections and placed in 300 cm^3^ Erlenmeyer flasks. Feathers were then sterilized by the gassing method [13].

To set up different variants of the experiment, each medium with keratin substrate was inoculated with a two-week culture of the test strains. For this purpose, 10 cm^3^ of sterile distilled H_2_O with Tween 80 was added to tubes with *Trichophyton ajelloi* on slants with Sabouraud medium, and, after rinsing the spores and obtaining the starting suspension (10^−1^ cfu cm^−3^), a series of 1:10 dilutions was prepared. Subsequently, 1 cm^3^ of the prepared spore suspension with a density of 10^6^–10^8^ cfu cm^–3^ was evenly applied to feather surface in flasks with medium. 

#### 2.5.2. Optimization of Substrate Composition

The basal medium with the addition of 1% glucose or xylose was used to select an easily available source of carbon and energy. In order to determine the optimal concentration of keratin substrate, a mineral medium with 1.5% and 2.0% chicken feathers was used as a source of C, N, and S, in addition to the basal variant with 1% feathers. The cultures were carried out at 28 °C under static conditions. 

#### 2.5.3. Determination of Optimal Medium pH

The experiment was conducted on the basal medium under static conditions at 28 °C in a liquid mineral medium (with the composition as above), whose pH was adjusted to 4.5, 6.5, and 8.5. 

#### 2.5.4. Determination of Optimal Culture Temperature 

Culture of three selected *Trichophyton ajelloi* strains was carried out in the basal mineral liquid medium under static conditions with the addition of 1% feather waste using incubation temperature of 20, 28, and 37 °C.

#### 2.5.5. Determination of Optimal Culture Method

The experiment was conducted in two culture variants: static (surface cultures) and agitated (submerged cultures, 180 rpm/min, amplitude 5) (Water Bath Shaker, ELPIN_SC_+ type 357, ELPIN, Katowice, Poland) using the basal mineral liquid medium with 1% feather addition and a culture temperature of 28 °C.

### 2.6. Parameters of Trichophyton ajelloi Biodegradation Activity

The dynamics of changes in the activity of enzymes involved in the decomposition of waste feather keratin, concentration of mineral and organic forms of nitrogen and sulfur, and medium pH were periodically determined (i.e., after 7, 14, 21, 28, and 35 days) in the culture fluids of individual experimental variants.

#### 2.6.1. Determination of the Activity of Enzymes Involved Feather Keratin Biodegradation

Keratinase activity was measured using the method of Yu, et al. [37], modified by Anbu, et al. [38], in 0.1 M Tris–HCl buffer pH 8.1 using 20 mg of shredded chicken feathers as a substrate. Protease activity was determined by the method of Anson [39], modified by Korniłłowicz [40], in 0.028 M phosphate buffer pH 7.8, with the addition of 2 mM magnesium ions and 1% casein as a substrate. Determination of caseinolytic protease and keratinase activities was carried out using a UV–VIS–1800 spectrophotometer (RayLeígh, Beijing, China) at a wavelength of 720 and 280 nm, respectively. Determination of disulfide reductase activity was carried out spectrophotometrically (EPOCH^TM^ microplate spectrophotometer, BioTek, Winooski, VT, USA) according to Holmgren [41], modified by Aharanowitz, et al. [42], in 50 mM Tris–HCl buffer at pH 8.0, in the presence of NADPH and EDTA and 0.02 mM DTNB in ethanol.

#### 2.6.2. Determination of Organic and Mineral Forms of Nitrogen and Sulfur and pH

Free proteins and peptides in culture fluids were measured according to the method of Lowry [43], modified by Schacterle and Pollack [44]. Amino groups were determined by the ninhydrin method according to Korniłłowicz–Kowalska [30]. The content of ammonium ions in the culture fluids was determined by the nesslerization method [31], while sulfate content was determined based on the intensity of turbidity in the reaction with barium chloride (BaCl_2_) by nephelometry [31]. The concentration of thiol groups in the culture fluids was determined by color reaction of –SH groups with Ellman’s reagent [45]. The pH of the culture fluids was determined using a CP–505 pH meter (Elmetron, Zabrze, Poland) equipped with a glass electrode. 

Amino acid composition, i.e., concentration of aspartate (Asp), threonine (Thr), serine (Ser), glutamate (Glu), proline (Pro), glycine (Gly), alanine (Ala), cysteine (Cys), valine (Val), methionine (Met), isoleucine (Ile), leucine (Leu), tyrosine (Tyr), phenylalanine (Phe), histidine (His), lysine (Lys), arginine (Arg), and tryptophan (Trp) of the optimized cultures tested were carried out according to the methodology described in detail by Bohacz, et al. [14]. For this purpose, culture fluids of the fungus strain III from 18-day optimized stationary cultures, with the addition of xylose and glucose, incubated at 20 and 37 °C, and from an agitated culture were selected. Amino acids were determined by ion exchange chromatography using an amino acid analyzer AAA 400 (Ingos, Prague, Czech Republic).

#### 2.6.3. Feather Weight Loss

Weight loss of the keratin substrate (feathers) was determined after 42 days of the experiment using the weighing method after drying at 105 °C.

### 2.7. Statistical Analysis

The results were statistically analyzed using Statistica ver.13.3 software (StatSoft, Kraków, Poland). A multivariate analysis of variance (ANOVA), followed by a post-hoc HSD–Tukey test was performed to demonstrate significant differences between the tested *Trichophyton ajelloi* strains in terms of changes in the pH of post-culture fluids, degree of keratin substrate degradation and differences in the ability of these strains to biodegrade chicken feather wastes at the α = 0.05 level of significance. One-way ANOVA followed by a post-hoc HSD-Tukey test (α = 0.05) was used to demonstrate differences in enzymatic indices of the examined *T. ajelloi* strains and differences between the dates of analyses of chicken feather biodegradation parameters.

Principal component analysis (PCA) was conducted, as described in the study of Nilsson, et al. [46], to demonstrate the relationship between keratinolytic activity of *Trichoderma ajelloi* fungal strains No. III, XII, and XIV and the optimized culture parameters. Indicators of keratinolytic activity included keratinase activity, protease, disulfide reductase, release of soluble proteins and peptides, amino and thiol groups, ammonium and sulfate ions, as well as changes in pH and feather weight loss. Optimized culture parameters included readily available carbon and energy source (glucose—G, and xylose—X), different amounts of feathers (1.0, 1.5 and 2.0% feather waste), culture temperature (20, 28 and 37 °C), baseline pH of the medium (4.5, 6.5, 8.5), culture method (stationary—ST, agitated—AG). The results of the analysis are presented on the score plot and loading plot. The score plot (dots) and loading plot (vectors) should be interpreted simultaneously [46]. Two plots help analyze correlations between observations and variables. The loading plot shows the effect of variables, i.e., keratinase activity, protease, disulfide reductase, concentration of soluble proteins and peptides, amino and thiol groups, ammonium and sulfate ions, as well as pH and feather weight loss in individual principal components and demonstrates the correlation structure relative to the variables. The score plots is a map of observations and represents optimization parameters. If the observation on the score plot (different optimized parameters in cultures of different strains) is in the corresponding part on the loading plot, the influence of the variable on the observation is greater.

Based on the analysis of variance, the optimal conditions for the most effective biodegradation of chicken feather waste by *Trichophyton ajelloi* strains were proposed for all optimized parameters of keratin biodegradation.

## 3. Results

### 3.1. Preliminary Evaluation of Temperature Effect on Trichophyton ajelloi Growth

A preliminary evaluation of the effect of temperature on the linear growth of the studied *Trichophyton ajelloi* strains on Sabouraud and MAE (maltose medium) at four different temperatures (20, 28, 37, and 50 °C) showed a lack of growth of these fungi at 37 and 50 °C. It was observed that Sabouraud medium (high glucose) promoted the growth of *T. ajelloi* fungi. This was expressed as a 24% larger colony diameter after 7 and 14 days of culture (Table 1). This suggested an introduction of an additional readily available source of carbon and energy for fungal culture into the optimization process.

### 3.2. Preliminary Evaluation of Hydrolytic Abilities of Trichophyton ajelloi 

The initial assessment of *Trichophyton ajelloi* fungi for the degradation of complex organic matter, i.e., protein, fat, and starch was evaluated on the basis of the calculated enzymatic index (IE). Strain No. III showed the greatest, but not significantly different (*p* < 0.05) fat degradation capacity, while strain No. XIV showed a significantly higher ability to degrade protein in comparison to two other *T. ajelloi* strains (Table 2).

### 3.3. Optimization of Culture Conditions and Substrate Composition

In order to select optimal culture parameters for the biodegradation process of native keratin from chicken feather by geophilic strains of *Trichophyton ajelloi*, different medium pH, incubation temperature, different amounts of chicken feathers, additional carbon sources and different culture methods were tested. The process of optimizing keratin biodegradation was evaluated in terms of measuring the activity of keratinase, protease, disulfide reductase, concentration of released soluble proteins and peptides, total pool of amino acids, as measured by the content of amino and thiol groups, as well as ammonium and sulfate ions, changes in medium pH and feather weight loss.

#### 3.3.1. Effect of Carbon and Nitrogen Source on Feather Keratin Biodegradation by *Trichophyton ajelloi*

##### Enzymatic Activity

Protease, keratinase, and disulfide reductase activities were determined in the selection of the optimal carbon source for the biodegradation of native feather keratin by *Trichophyton ajelloi* fungal strains. Based on the results, it was found that all strains tested in the presence of glucose and xylose carry out the process of feather keratin biodegradation. The activity of keratinase and caseinolytic protease in cultures of all strains labeled as III, XII, and XIV was more efficient in the presence of glucose than xylose. Keratinase activity remained high throughout the experiment and gradually increased, especially in strain III cultures. Protease activity increased significantly for the first 2 weeks of the experiment, and subsequently decreased slightly and again significantly increased from day 28 both in cultures with glucose and xylose addition. The maximum activity of this enzyme in cultures with glucose was on day 14 and in cultures with xylose on day 35. Compared to baseline, the activity of this enzyme increased by 311% in cultures with glucose and by 335% in cultures with xylose, respectively. The addition of xylose in the first 14 days had an inhibitory effect on keratinase activity. At the end of the experiment, both xylose and glucose stimulated the activity of this enzyme. 

**Figure 1:**
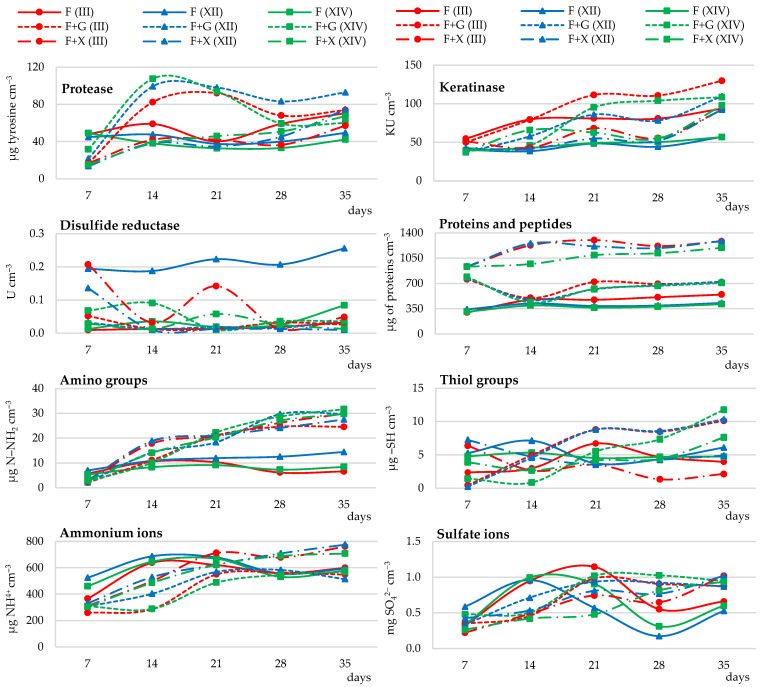
Dynamics of changes in enzymatic activity and released organic and mineral products of keratinolysis in cultures of three *Trichophyton ajelloi* strains III, XII, and XIV, with the addition of various easily available sources of carbon and energy, i.e., glucose (G) and xylose (X).

Keratinase activity at the end of the experiment was on average 177% higher compared to the initial time point in the glucose-supplemented cultures and 115% higher in the xylose-supplemented cultures. In cultures of *Trichophyton ajelloi* strain III, keratinase activity was stimulated by the presence of xylose only in the last experimental time point (Figure 1 and Supplementary Table S1). Disulfide reductase activity in *Trichophyton ajelloi* cultures generally declined until day 14, followed by its increase and then another decrease after day 28, especially in cultures with xylose. Disulfide reductase activity was on average higher than the activity of this enzyme in cultures without the addition of a readily available carbon and energy source by an average of 42% and 121% in cultures with glucose and xylose addition respectively, with the exception of strain XII cultures. The highest activity of this enzyme throughout the experiment was recorded in cultures of strain XII (Figure 1 and Supplementary Table S1).

##### Release of Soluble Proteins, Peptides and Amino Acids

The introduction of xylose into the culture medium resulted in a higher release of soluble proteins and peptides, on average 43% higher than in the presence of glucose and without the additional carbon and energy source, especially in the culture of strains III and XII. The total pool of amino acids, as measured by the concentration of amino groups, was clearly affected by the addition of both sugars glucose and xylose. The release of amino groups until day 21 of the culture of all tested fungal strains was stimulated by xylose and from day 21 of the culture by glucose. In the cultures without glucose and xylose, amino groups were released in greater quantities during the first week of the experiment. These quantities were on average higher by 169% and by 119% compared to cultures with glucose and xylose, respectively. The number of these groups starting from day 21 of the culture decreased or remained constant. It was shown that the pool of sulfur amino acids, measured by the release of thiol groups, up to day 7 of the culture was induced by the presence of xylose and from day 21 of the experiment by glucose, especially in the cultures of strains III and XII. This was evidenced by the significantly higher concentrations of these groups in culture fluids on average by 45% compared to culture with xylose (Figure 1 and Supplementary Table S1).

##### Ammonium Ion Release and Sulfate Ion Generation

The addition of glucose and xylose to the cultivation of all fungi resulted in a gradual increase in the release of ammonium ions. In relation to the whole experiment, 1% xylose was more effective (by 29%) in stimulating the secretion of ammonium ions than 1% glucose. Ammonium ion release during feather biodegradation in cultures without the addition of glucose and xylose remained high for all fungal strains for 21 days of the culture, after which it decreased and increased again after 28 days of the culture. Compared to 21-day cultures with glucose and xylose, the release of these ions was on average 22% and 1% higher, respectively. In the culture fluids of strains XII and XIV, the beneficial effect of the addition of 1% xylose on the release of ammonium ions became apparent after 28 days of analysis. A strong stimulating effect on sulfate release was demonstrated in the cultures with glucose, especially after 21 days of the culture. Compared to the cultures with xylose, sulfate generation after this time was on average higher by 44%. In contrast, it was lower after the application of 1.0% xylose. It was also shown that sulfate release by all tested fungal strains in the cultures not enriched with sugars was high until day 21 of the culture, then decreased by an average of 67% compared to the maximum concentration, and increased again after day 28 of the culture (Figure 1 and Supplementary Table S1).

##### Effect of Chicken Feather Waste Concentration on Biodegradation Activity of *Trichophyton ajelloi*

**Figure 2:**
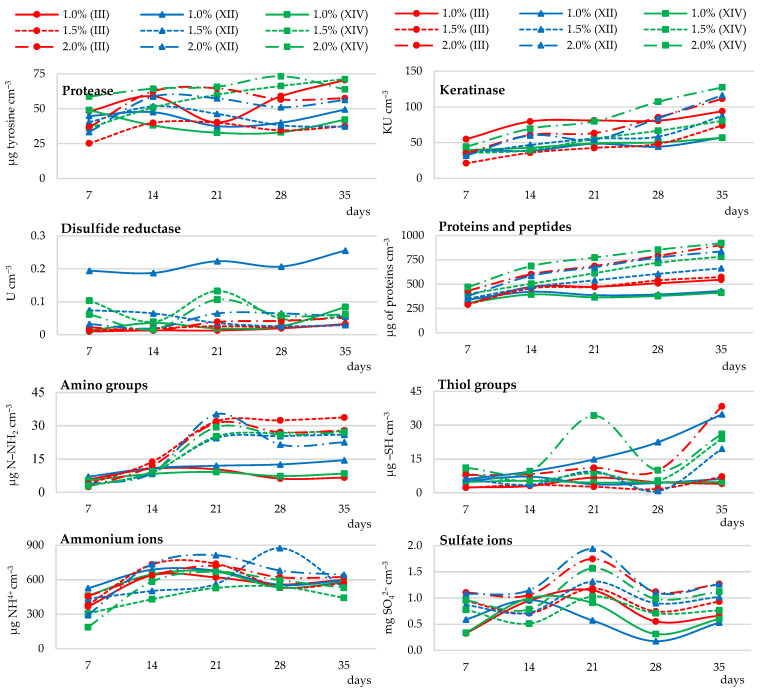
Dynamics of changes in enzymatic activity and released organic and mineral keratinolysis products in different concentrations of feather waste analyzed in cultures of three *Trichophyton ajelloi* strains III, XII, and XIV.

Optimization of culture conditions with regard to the content of the hardly accessible source of C, N, and S, i.e., native keratin involved three feather doses of 1, 1.5, and 2%. It was shown that higher feather doses generally stimulated all microbial biodegradation parameters. In particular, 2% content of keratin material, compared to 1% basal feather concentration, resulted in a significant increase in the secretion of soluble proteins and peptides by an average of 69%, sulfates by 89%, and thiol groups by an average of 255% into the medium, as well as keratinase, protease activities, especially in cultures of strains XII and XIV (by an average of 66%, and 41%, respectively. Disulfide reductase activity also increased especially in cultures of strains designated No. III and XIV. Sulfate release was clearly linked to disulfide reductase activity, especially on day 21 of the culture, as well as the release of amino groups (Figure 2 and Supplementary Table S2).

##### Changes in the pH of Culture Fluids

Measurements of the pH of culture fluids from the cultures enriched with glucose and xylose indicated an increase in pH up to day 28 of the culture and subsequent stabilization. The values of this index in the variant with glucose were in the range of 6.90–8.86 (strain No. III), 7.10–8.93 (strain No. XII), 7.03–8.88 (strain No. XIV), and with xylose in the range of 6.93–8.64 (strain No. III), 7.00–8.64 (strain No. XII), and 6.70–8.61 (strain No. XIV). Increasing the amount of chicken feather waste resulted in an increase in the pH of culture fluids, up to day 14 of the culture and subsequent stabilization. In the cultures with 1.5% feathers, the pH ranged from 8.53 to 9.13 (strain No. III), 8.48–9.21 (strain No. XII), 7.24–8.75 (strain No. XIV), and with 2% feathers from 8.13 to 8.95 (strain No. III), 8.54–9.32 (strain No. XII), and 7.84–8.99 (strain No. XIV) (Table 3).

##### Feather Weight Loss

The addition of glucose to cultures containing native feathers resulted in a reduced degradation rate of this substrate compared to cultures containing feathers as the only carbon and energy sources. This loss in cultures with 1% feather and glucose addition was estimated at 6%. Slowdown in feather biodegradation with respect to 1% feather cultures not enriched with sugars amounted to 83%. The highest feather weight loss (58% on average), significantly higher compared to other conditions (1.0 and 1.5%), was demonstrated in the cultures without the addition of glucose and xylose, containing 2% feather waste (Table 4). An increase in the concentration of chicken feathers in fungal cultures increased the weight loss of this substrate by an average of 64%.

#### 3.3.2. Effect of Culture Medium pH on Biodegradation of Chicken Feathers

##### Enzymatic Activity

**Figure 3:**
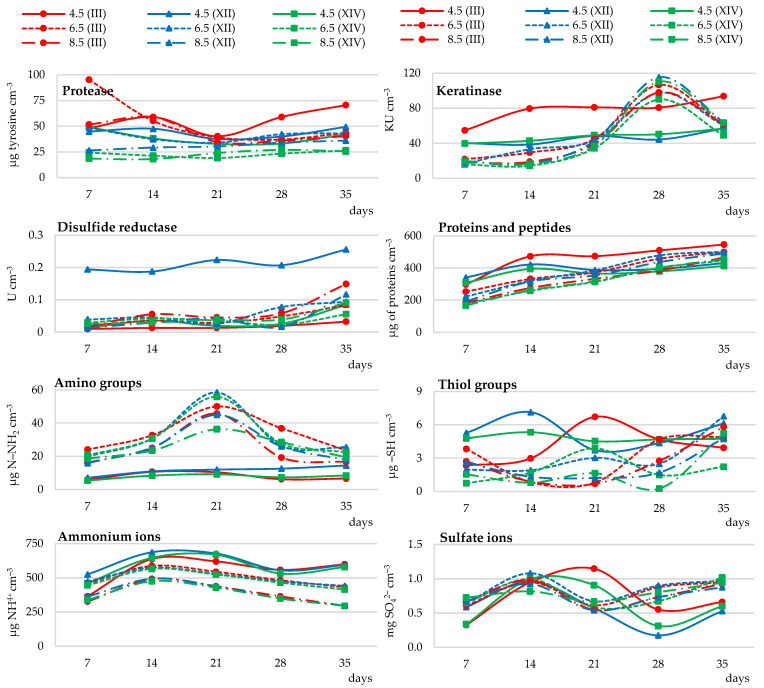
Dynamics of changes in enzymatic activity and released organic and mineral keratinolysis products in different initial medium pH values analyzed in cultures of three *Trichophyton ajelloi* strains III, XII, and XIV.

The dynamics of chicken feather biodegradation by geophilic strains of *Trichophyton ajelloi* were analyzed in the pH range of 4.5 to 8.5 in 1.0 unit increments, using a culture temperature of 28 °C and 1% feather addition. It was found that all strains tested had the ability to biodegrade keratin in the analyzed pH ranges. However, they differed in the intensity of the process. Up to day 21 of the culture, keratinase activity of all fungal strains in each of the tested variants was low, but steadily increased, reaching a maximum on day 28 of the culture, especially at the initial pH of 8.5. At this time point, keratinase activity was on average 85% higher compared to the activity of this enzyme at pH 4.5. On the other hand, protease activities of the tested strains in general decreased until day 21 of the culture and then increased. Protease activity in cultures with lower initial pH was on average 17% and 41% higher compared to cultures with pH 6.5 and 8.5. At the lowest initial pH, i.e., 4.5, keratinase activity increased throughout the experiment and was the highest in the culture of strain III. Disulfide reductase activity increased significantly, starting from day 28 of the culture. This effect became apparent in the cultures of all strains tested and was independent of the initial medium pH. The highest activity of this enzyme was recorded in the culture of strain XII with an initial medium pH of 4.5. It was higher than the activity in the other experimental variants by an average of 80% (Figure 3 and Supplementary Table S3).

##### Release of Soluble Proteins, Peptides, and Amino Acids

The release of soluble proteins and peptides significantly increased in the cultures with higher baseline pH (6.5 and 8.5), while it generally decreased when the initial pH was 4.5, especially in relation to strains XII and XIV. This was an average increase of 122% at pH 6.5 and 160% at pH 8.5 compared to the initial time point. The total pool of amino acids, as measured by N amino (N–NH_2_) content, was 88% higher in the cultures with higher baseline pH compared to cultures with initial pH of 4.5. The increase in amino group content was strongest in the cultures with an initial pH of 6.5. The maximum release of amino groups, occurred on day 21 of the culture of the tested fungal strains. Increasing the pH of the culture medium, on the other hand, reduced the release of thiol groups in the cultures of *Trichophyton ajelloi* strains by an average of 42% at pH 6.5 and 52% at pH 8.5, and the dynamics of their release varied depending on the culture time and fungal strain (Figure 3 and Supplementary Table S3).

##### Ammonium Ion Release and Sulfate Ion Generation

Secretion of mineral forms of nitrogen, i.e., ammonium ions was stimulated most strongly in cultures with an initial medium pH of 4.5 and was the weakest at an initial pH of 8.5. The content of ammonium ions in cultures with an initial pH of 4.5 was, on average 17% higher compared to the content of these ions at pH 6.5 and by 52% at pH 8.5. By day 14 of the culture, a significant increase in NH_4_^+^ concentration in the medium was recorded. After this time point, there was a slow but significant decrease in the concentration of ammonium ions in the cultures of all 3 strains, persisting until day 28 of the experiment. Sulfate secretion in the tested pH variants significantly increased until day 14 of the culture of all tested fungi. It was on average higher at baseline pH of 6.5 and 8.5 by 27% and 20%, respectively, compared to 4.5. Between days 14 and 21 of the culture, SO_4_^2−^ ion content decreased and then increased significantly until day 35 of the culture. Sulfate production was stimulated most strongly in cultures at pH = 6.5. The exceptions were cultures of fungi III and XVI at pH = 4.5, in which the concentration of these ions increased until day 21 of the culture, transiently decreased, and from day 28 of the culture increased again (Figure 3 and Supplementary Table S3). 

##### Changes in Medium pH

It was shown that pH of the culture media in the first week of the experiment, regardless of the initial pH value, increased to approx. 8.0. Culture fluid pH in the variants with baseline pH of 6.5 and 8.5 was the highest and remained stable until day 21 of the culture, after which it slightly decreased. For variants with an initial pH = 6.5, the pH values ranged from 8.42 to 9.24 (strain No. III), 7.74–8.86 (strain No. XII), 8.45–9.18 (strain No. XIV), and for variants with an initial pH = 8.5—8.61–9.32 (strain No. III), 8.36–9.08 (strain No. XII), 8.68–9.28 (strain No. XIV) (Table 3).

##### Feather Weight Loss

The degree of chicken feather waste decomposition was the highest when the initial medium pH was the lowest (pH = 4.5). It was 37% in cultures of all fungal strains incubated under these conditions (Table 4). The strongest solubilization of feathers was recorded for strain III. In most experimental variants, the highest but statistically insignificant feather weight loss was recorded in cultures of this *Trichophyton ajelloi* strain. Increasing medium pH to 8.5 reduced the efficiency of decomposition of chicken feather waste by 27% (Table 4). 

#### 3.3.3. Effect of Culture Medium Temperature on Chicken Feather Biodegradation

##### Enzymatic Activity

**Figure 4:**
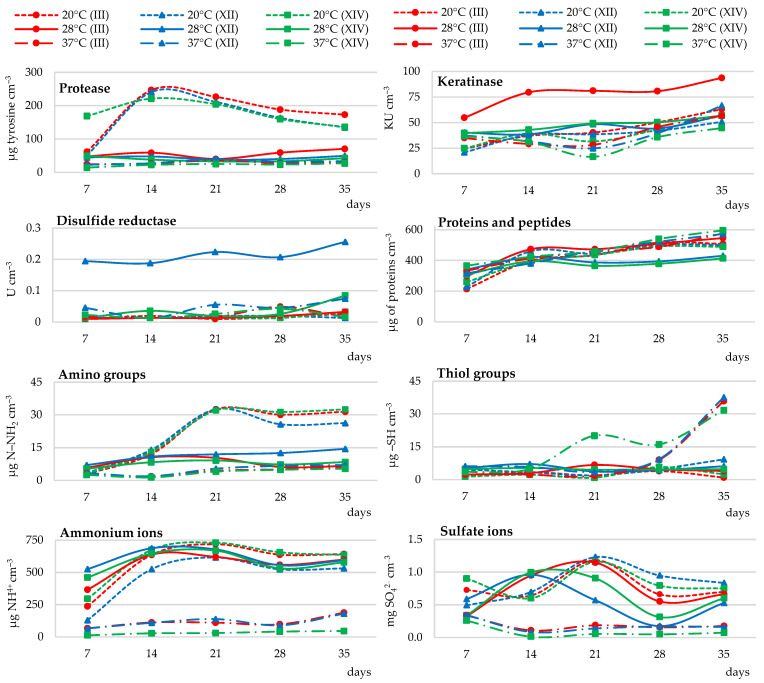
Dynamics of changes in enzymatic activity and released organic and mineral keratinolysis products in different culture temperatures of three *Trichophyton ajelloi* strains III, XII, and XIV.

*Trichophyton ajelloi* strains at all three temperatures tested differed in the biodegradation activity of chicken feather waste. It was shown that a temperature of 20 °C promoted their proteolytic activity. This activity was higher, on average by 79%, than the activity of this enzyme in culture fluids collected from cultures incubated at 28 °C and 37 °C. Caseinolytic protease activity in each temperature variant increased until day 14 of the culture, after which a gradual decrease was recorded especially in cultures incubated at 20 °C. In cultures incubated at 28 and 37 °C, the activity of this enzyme increased again after day 21 of the culture. Keratinase activity was the highest in strain III cultures at 28 °C and was on average higher by 48% throughout the study period compared to other variants. Increasing the culture temperature did not significantly stimulate an increase in the activity of this enzyme as did disulfide reductase activity. Disulfide reductase activity was the highest in cultures of strain XII growing at 28 °C. This activity was on average 456% and 187% higher than the activity of this enzyme in cultures incubated at 20 °C and 37 °C, respectively. Temperature of 37 °C slightly stimulated disulfide reductase activity starting from day 21 of the culture (Figure 4 and Supplementary Table S4).

##### Release of Soluble Proteins, Peptides, and Amino Acids

The release of soluble proteins and peptides increased steadily throughout the culture of all fungal strains at all temperatures. The lowest concentrations of these feather decomposition products were recorded in cultures incubated at 28 °C, especially after day 14 of culture. The highest accumulation of soluble proteins and peptides was recorded in the last week of the experiment when the incubation temperature was 37 °C. Amino acid content, as measured by the concentration of amino groups, increased until day 21 of the culture at 20 °C and remained at the same, stable level until the end of the experiment. On average, it was 58% higher than the content of these compounds in cultures conducted at 28 °C and 80% higher than at 37 °C. The overall concentration of sulfur amino acids, as measured by the level of thiol groups in the medium, was the highest in cultures incubated at 37 °C especially after day 21 of the culture (Figure 4 and Supplementary Table S4).

##### Ammonium Ion Release and Sulfate Ion Generation

The concentration of ammonium ions in all fungal cultures, incubated at three different temperatures, increased until day 14 of the culture. After this time, the release of these ions continued to raise in cultures incubated at 20 and 37 °C until day 21 of the culture. At the same time, it was higher than the cultures incubated at 37 °C by an average of 60%. The concentration of sulfate ions increased for the first 14 days of the culture, reaching the highest level in cultures incubated at 28 °C. Later, only the temperature of 20 °C had a stimulating effect on the secretion of sulfate ions in the cultures of all 3 fungal strains. Accumulation of ammonium and sulfate ions was the lowest in cultures incubated at 37 °C. In comparison to 20 °C, this was on average 84% and 82% lower, respectively (Figure 4 and Supplementary Table S4).

##### Changes in the pH of Culture Fluids

In all temperature variants, the pH of the cultures increased to values above 6.0 during the first week of the experiment. The increase in pH was the highest in cultures incubated at 20 °C, while the lowest in cultures incubated at 37 °C. This corresponded to pH values ranging for a temperature of 20 °C from 7.76 to 9.10 (strain No. III), 6.99–8.92 (strain No. XII), 8.08–9.08 (strain No. XIV), and 6.39–6.92 (strain No. III), 6.35–6.67 (strain No. XII), and 6.05–6.20 (strain No. XIV) for 37 °C. Slight changes in pH were also recorded in cultures incubated at 28 °C in the range of 7.81–8.90 (strain No. III), 8.51–8.79 (strain No. XII) and 8.44–8.82 (strain No. XIV) (Table 3). 

##### Feather Weight Loss

Significantly higher feather weight loss due to keratin biodegradation, averaging 37%, was recorded in cultures incubated at 28 °C. In fungal cultures incubated at 37 °C, it ranged from 17 to 21%. With respect to the highest feather weight loss, it was an average reduction in biodegradation efficiency of 48% (Table 4).

#### 3.3.4. Effect of Culture Method on Chicken Feather Biodegradation

##### Enzymatic Activity

**Figure 5:**
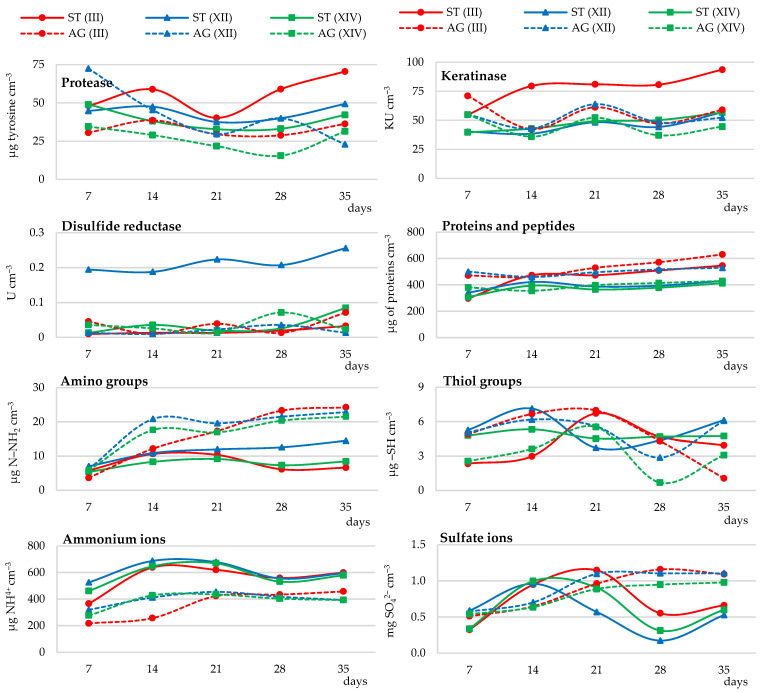
Dynamics of changes in enzymatic activity and released organic and mineral keratinolysis products in different culture methods (agitated—AG and stationary—ST) analyzed for three *Trichophyton ajelloi* strains III, XII, and XIV.

It was found that there was a decrease in proteolytic activity in agitated cultures of all strains tested. This trend was evident across all analysis time points. On the other hand, keratinase activity was stimulated by culture agitation, especially up to day 21 of incubation. This particularly concerned strains III and XII. In addition, it was shown that protease activity was the lowest in all cultures on day 21 of fungal growth, when keratinase activity was high. Both activities were significantly the highest in cultures of strain III grown under static conditions in mineral medium with 1% feathers, pH = 4.5 and an incubation temperature of 28 °C. Keratinase and protease activities were higher than the activity of these enzymes in agitated cultures of this fungus strain by an average of 39% and 69%, respectively (Figure 5 and Supplementary Table S5).

##### Release of Soluble Proteins, Peptides and Amino Acids

Conducting submerged cultures generally had a favorable effect on the release of soluble proteins and peptides by the studied fungi. Their concentration steadily increased during the culture. This was an average increase of 18% in agitated cultures and 47% in stationary cultures compared to the initial time point. Significantly higher content of protein products was recorded in the culture of strain III (Figure 5 and Supplementary Table S5). The content of amino acids in these cultures, measured by amino group concentration, was significantly higher compared to the stationary cultures by an average of 88%. The concentration of NH_2_ groups in the cultures of all 3 fungal strains increased until day 35 of culture, reaching a significantly higher level than at earlier time points by an average of 62% and 337% in stationary and agitated cultures, respectively. The stimulating effect of the release of thiol groups in the submerged cultures was detected at the initial and final time points of the culture, i.e., until day 21 and after day 28 for all fungal strains. On the other hand, a reduction in the ability of the tested strains to release–SH groups was noted between days 21 and 28 of incubation with agitation (Figure 5 and Supplementary Table S5).

##### Ammonium Ion Release and Sulfate Ion Generation

The release of ammonium ions in the agitated cultures was significantly lower by an average of 34% compared to the stationary cultures at all experimental time points of the three tested strains. The release of these ions in both culture variants increased until day 21, and then decreased. Their highest concentration was recorded in the culture of strain XII. Accumulation of sulfate ions in the agitated cultures was particularly pronounced after day 21 of the culture. With respect to non-agitated cultures, the concentration of these ions gradually increased until day 21 and after day 28 of the culture (Figure 5 and Supplementary Table S5). 

##### Changes in the pH of Culture Fluids

Culture fluids from agitated cultures had lower pH values by day 21 of the culture. After this time, the pH of the fluids remained constant in both types of the culture. The reported pH values ranged from 7.68 to 8.76 (strain No. III), 8.23–8.98 (strain No. XII), 8.19–8.93 (strain No. XIV) in agitated cultures, and 7.81–8.90 (strain No. III), 8.51–8.79 (strain No. XII), 8.44–8.82 (strain No. XIV) in stationary cultures (Table 3).

##### Feather Weight Loss

Agitation of the culture caused a significant decrease in the efficiency of chicken feather biodegradation by the tested *Trichophyton ajelloi* strains, on average by 52%. This corresponded to a feather weight loss of 16–22% (Table 4).

#### 3.3.5. Amino Acid Composition in Optimized Cultures

The analysis of amino acid composition of 18-day culture fluids of the *T. ajelloi* strain III showed that tryptophan was the most abundant amino acid in almost all culture fluids, with the exception of culture fluid with 1.0% glucose, where proline was the dominant amino acid. Aspartate and cysteic acid were also abundant. The culture fluid with 1.0% xylose not only had the highest content of tryptophan but also of methionine sulfonate. Of all the culture fluids analyzed, medium from the culture incubated at 37 °C was characterized by increased levels of all tested amino acids except proline, cysteic acid, and methionine sulfonate. The fluid from agitated culture contained a high quantity of isoleucine and histidine in its composition. The richest source of amino acids was determined in the fluid from *Trichophyton ajelloi* cultures incubated at 37 °C, while the poorest was the fluid from cultures at 20 °C (Table 5). 

### 3.4. Statistical Evaluation of Culture Condition Optimization 

**Figure 6:**
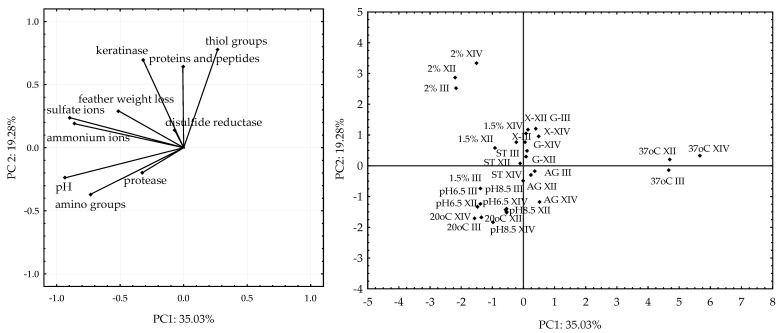
PCA loading plot and score plot demonstrating relationships between fungal keratinolysis indicators, i.e., enzymatic activity, concentration of soluble proteins, peptides, amino acids, ammonium and sulfate ions, as well as feather weight loss, different culture conditions, and medium composition in *Trichophyton ajelloi* cultures.

The obtained results, subjected to principal component analysis in order to explain the relationship between the indicators of fungal biodegradation of post-production chicken feathers and the optimized parameters of fungal culture, showed that the applied model explained 54.31% of the variability by two principal components (PC1: 35.03% and PC2: 19.28%). The loading plot (Figure 6) shows the correlation structure of the analyzed variables, i.e., protease, keratinase and disulfide reductase activities, concentration of soluble proteins and peptides, amino and thiol groups, and ammonium and sulfate ions, pH changes and feather weight loss. Location of the dots on the score plot grouped optimized breeding conditions, i.e., an easily accessible source of carbon and energy, different amounts of chicken feathers, different initial pH of the medium, temperature, and breeding method in relation to the parameters (variables) tested. The results demonstrated the association of keratinase activity, feather weight loss, and sulfate and ammonium ion concentrations with high content of chicken feather waste (1.5 and 2%) (observation dots). In addition, this analysis indicated significantly positive correlations between these variables. The release of amino groups, changes in pH and protease activity (variables on the loading plot) depended mainly on the higher initial pH, i.e., 6.5 and 8.5, and on culture at 20 °C (dots on the score plot). The concentration of soluble proteins and peptides and thiol groups significantly depended on the presence of an easily available carbon and energy source, i.e., xylose and glucose. The significantly positive correlation between these variables and keratinase activity indicated that this enzyme played a crucial role in the release of organic breakdown products of native keratin (soluble proteins and peptides, as well as amino and thiol groups) and indirectly also inorganic sulfur compounds (sulfates). Based on the presented data, it could be concluded that protease activity, compared to keratinase, was less important in the process of feather keratinolysis by the tested fungal strains, and the temperature of 37 °C was not conducive to their biodegradation activity (Figure 6).

The multivariate ANOVA (Table 6) showed significant differences between *T. ajelloi* strains in the release dynamics of individual keratinolysis products and keratinolytic enzyme activity in individual optimization variants. Analyzing the results, it can be concluded that the significantly different and highest parameters of keratinolytic activity, measured by keratinase and protease activity under optimized conditions concerned strain No. III (Table 6). The release of high concentrations of keratinolysis organic products, i.e., soluble proteins, peptides, and amino acids, as measured by the content of amino and thiol groups, was significantly different in cultures of strains III and XII. It was also shown that strain XII, in contrast to other *Trichophyton ajelloi* strains, showed the highest mineralization abilities. This was expressed in significantly higher concentrations of ammonium and sulfate ions in most of the optimized cultures compared to the remaining strains. This is important from a fertilization point of view and translates into the future use of this strain in obtaining feather hydrolysates (biopreparations) with biofertilizer properties (Table 6).

**Figure 7:**
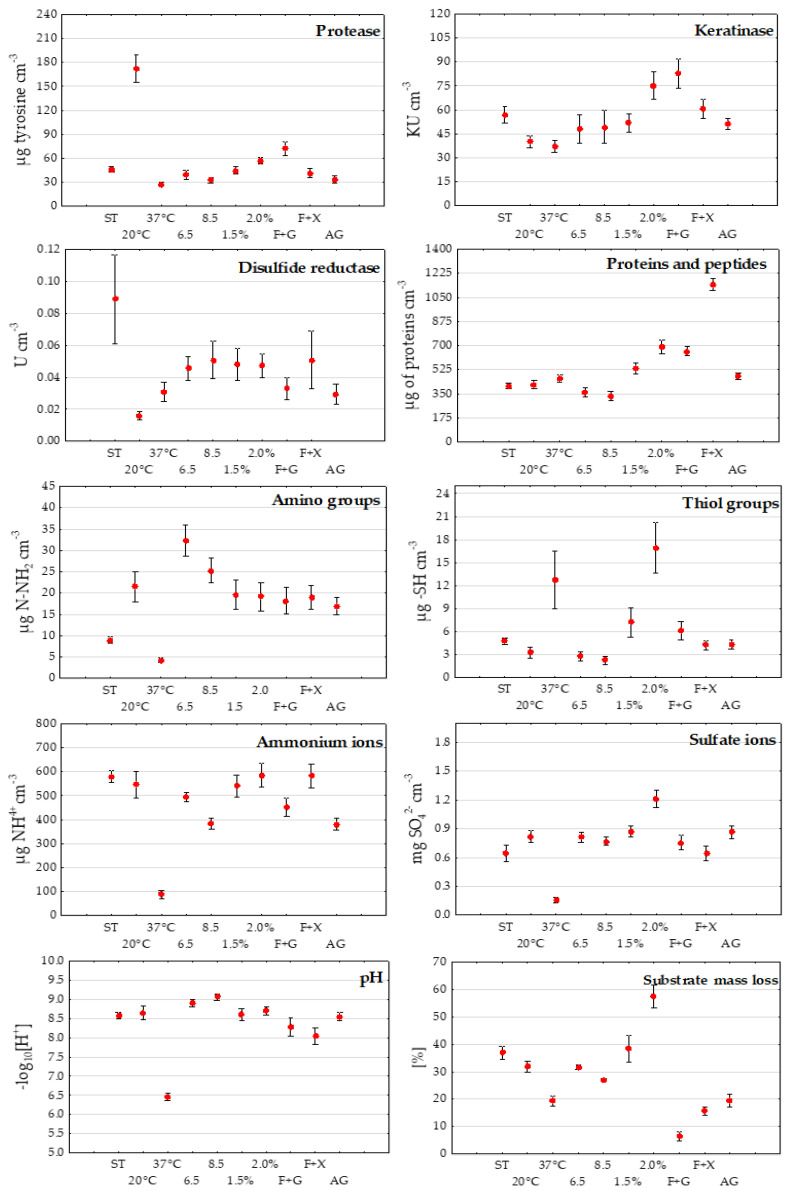
Summary of significant differences within the analyzed cultivation conditions of *Trichophyton ajelloi*; α = 0.05.

Testing different culture conditions and medium composition to increase the efficiency of *T. ajelloi* strains regarding the biodegradation of post-production chicken feathers for practical purposes led to several concluding observations: • For all fungal strains tested, the most optimal conditions for high protease activity occurred in culture medium with 1% glucose, 1% chicken feathers, pH 4.5, incubation temperature of 28 °C, and without glucose but at 20 °C; • For keratinase activity, the most optimal conditions, significantly different from the other optimized parameters, were provided by cultures with and without glucose with 1% chicken feathers, pH 4.5, and an incubation temperature of 28 °C, as well as in the presence of 2% chicken feathers; • Disulfide reductase activity was significantly the highest in stationary cultures with 1% chicken feathers, pH 4.5 and an incubation temperature of 28 °C; • Release and accumulation of soluble proteins and peptides in stationary cultures with the addition of 1% feathers and initial pH of 4.5 was enhanced by the addition of simple C and energy sources, mainly xylose, and incubation temperature of 28 °C; • Increasing feather concentration to 2% and glucose addition stimulated the release of higher amounts of these readily hydrolyzable N organic bonds; The release of amino acids, as measured by the concentration of amino groups, was generally favored by all culture conditions and medium composition, but most highly by starting pH of 6.5, 1% feathers, and incubation temp. of 20 and 28 °C; • Thiol groups were released efficiently in cultures without glucose and xylose with 2% feathers at substrate pH of 4.5 and an incubation temp. of 28 °C, and with the addition of glucose and 1% feathers at 37 °C; • Sulfate ion release was stimulated by all culture conditions (except 37 °C), in particular the addition of 2% chicken feathers, temperature of 28 °C and an initial medium pH of 4.5; Concentration of these compounds was recorded at an average of 1.21 mg cm^−3^; • Ammonium ions accumulated in the highest concentration in cultures conducted in the basal control medium (average 0.58 mg cm^−3^); • Feather decomposition, as measured by the loss of this substrate, was the highest in the medium with the addition of 2% feathers, pH 4.5 and incubation temperature of 28 °C (Figure 7).

## 4. Discussion

The subject of this study concerned the assessment of geophilic *Trichophyton ajelloi* strains, little known in terms of native keratin decomposition, for their utilization in feather waste management, along with a proposal to optimize the biodegradation conditions of these wastes, enabling obtaining biopreparations of high fertilizing quality.

The optimized medium composition and culture conditions involved the use of an additional readily available carbon and energy source, different feather concentrations, different temperature and pH of the medium, and the culture method. These included optimization of the secretion of enzymes active in keratinolysis, i.e., protease, keratinase and disulfide reductase, products of native keratin decomposition, i.e., soluble proteins and peptides, the total pool of amino acids, sulfate and ammonium ions, and the loss of feather mass.

According to Liang, et al. [47], the composition of the medium and culture conditions were very important in the process of keratinolytic enzyme production by fungi. In our preliminary experiments, the selected geophilic fungal strains of *Trichophyton ajelloi* showed better growth at 28 °C on Sabouraud medium rich in readily available C and energy than MEA medium, while they did not grow at 37 and 50 °C. Preliminary characterization of the hydrolytic properties of *Trichophyton ajelloi* fungi, expressed as the enzymatic index, has demonstrated that these fungi have a high protein degradation capacity, lower fat degradation capacity and a lack of amylolytic abilities, similar to other keratinolytic fungi from the dermatophyte group [48] and *Chrysosporium keratinophilum* [49]. Kunert [48] have reported, however, that while lipolytic abilities of keratinomycetes are common, there are exceptions with regard to amylolytic activity and some dermatophytes also show such abilities. However, they are considered rudimentary [48].

### 4.1. Feather Weight Loss

Bohacz [12] defined the loss of feather waste as a reliable indicator of the management and biodegradation of post-production feathers by environmentally friendly approach, i.e., biological methods using microorganisms, and defined it as an “economic coefficient.” Optimization of culture conditions showed that the tested *Trichophyton ajelloi* strains most effectively degraded feather waste at its 2.0% concentration. Moreover, temperature of 28 °C, an initial medium pH = 4.5, static culture conditions, and a lack of readily available carbon sources were favorable conditions for this substrate decomposition. Substrate degradation after 42 days of growth in these cultures was 57, 59, and 56% for strains No. III, XII, and XVI, respectively. Biodegradation efficiency of native feather keratin by the tested strains of *Trichophyton ajelloi*, as measured by keratin substrate weight loss, was similar or lower than that of other keratinolytic fungal species previously studied by Bohacz, et al. [14] and Bohacz [12]. The cited works showed that fungi of the species *Aphanoascus keratinophilus* and *Chrysosporium tropicum*, isolated from pellets of predatory birds, decomposed in static cultures containing native feather waste as the only source of C and energy, during the same time (42 days), from 44 to 76% of native feather keratin mass, while *Aphanoascus fulvescens*, isolated from soil, 50–65% of the mass of this substrate. Some keratinolytic fungi, such as those isolated from soils in rook colonies (*Arthroderma tuberculatum* and *Arthroderma multifidum*), decomposed from 27 to 34% of the weight of native chicken feathers already after 28 days of growth in stationary cultures [14]. Korniłłowicz–Kowalska [30] noted that the environment of origin may be one reason for the differences in keratinolytic activity of fungi. The latter author showed that fungi derived from acidic soils were characterized by a relatively lower keratinolytic activity than fungi isolated from neutral and alkaline soils. Perhaps the reason for the slightly lower keratinolytic activity of *T. ajelloi* strains compared to the strains of *Aphanoascus* spp. and *Chrysosporium* spp. was their habitat: Cambisol pH = 5.4 for *Trichophyton ajelloi* strains [26], and Phaeozem pH = 7.28 for *Aphanoascus fulvescens* and *Chrysosporium articulatum* strains [12], and pellets of birds of prey with a pH in the range of 5.93–7.50 for *Aphanoascus fulvescens* and *Chrysosporium tropicum* [13]. 

Modification of breeding conditions may accelerate or limit the weight loss of feather waste. Călin, et al. [50] reported that the average weight loss of feathers subjected to additional high-temperature thermal exposure, i.e., containing denatured keratin, in cultures of fungi of the genera *Trichophyton* and *Chrysosporium* was about 75% after 21 days. In turn, in this study, the biodegradation of feather keratin, measured by the loss of feather mass, was significantly limited by the addition of glucose to fungal cultures. The weight loss of feathers in these conditions was on average 5%, which could be explained by the utilization of an easily accessible source of C and energy to build mycelial biomass, which was very abundant in these cultures. In addition, agitated cultures (submerged growth) reduced the rate of chicken feather degradation by *Trichophyton ajelloi* strains by an average of 49%, and it ranged from 16.0 to 22.0%. Research by Muhsin and Hadi [51] showed that during 21 days of agitated cultures, *Trichophyton mentagrophytes* decomposed about 22% of feathers, *Microsporum gypseum* 7%, *Chrysosporium pannicola* 18%, and *Aspergillus flavus* as much as 32%.

### 4.2. Enzymatic Activity under Optimized Culture Conditions

According to Li [52], complete biodegradation of keratin requires an appropriate microorganism strain, properly optimized culture conditions and a keratinase-degrading system, which includes an enzyme mixture.

Considering the influence of easily available carbon and energy sources, i.e., glucose and xylose, on the overall keratinolysis process, including proteolysis, sulfitolysis, and keratinolytic attack, it was shown that the addition of glucose mainly stimulated protease and keratinase activity in the cultures of the three *Trichophyton ajelloi* strains. Similar results were obtained by Anbu, et al. [38], who showed high keratinase activity in cultures of *Trichophyton* sp. HA–2 in the presence of glucose, but complete inhibition in the presence of xylose. Kačinová, et al. [20] proved that introducing an easily digestible carbon source into the culture medium at a dose of 1.0% stimulated keratinolytic activity of *T. ajelloi*. The stimulating effect on glucose was significantly higher than that of sucrose, fructose and mannitol used by these scientists. The addition of easily digestible carbon and energy sources in the form of glucose or xylose to culture does not always increase keratinase activity. This applies in particular to the activity of these enzymes in cultures of non-keratinophilic strains, i.e., those not belonging to dermatophytes and fungi of the genus *Chrysosporium* specialized in native keratin degradation. This was demonstrated by Mini, et al. [53] and Anbu, et al. [54] in cultures of *Aspergillus flavus* and *Scopulariopsis brevicaulis* strains, who reported a decrease in keratinase activity in the presence of glucose and xylose, respectively. Anbu, et al. [54] and Singh [55] explained this effect by the catabolic repression of proteolytic enzymes by exogenous carbohydrates. Anbu, et al. [56] reported the accumulation of organic sulfur-containing end products as another reason for the inhibition of keratinase activity in fungal cultures. The temperature and pH of the culture, as well as substrate concentration were significant factors influencing the activity of keratinase and protease involved in the decomposition of native feather keratin. Optimization of keratinase and protease activity occurs at different temperatures and pH, as well as with different amounts of feather waste. Considering the effect of the concentration of chicken feather waste on the increased release of keratinolytic and proteolytic enzymes, El-Ghonemy and Ali [57] showed that higher production of keratinase and protease by the *Aspergillus* sp. strain DHE 7 was favored by increased native feather keratin concentration (2%). On the other hand, a further increase in feather concentration to 3, 4, and 5% inhibited keratinase activity, but not the release of soluble proteins and peptides. It is possible, as El-Ghonemy and Ali [57] assumed, that this effect was related to the activity of fungal proteases. As opposed to keratinases, whose secretion is induced by the presence of a substrate, proteases belong to constitutional enzymes and their secretion is induced by nutritional stress, i.e., the lack of carbon, nitrogen, and energy sources [58]. Anbu, et al. [54] reported that extremely acidic (pH = 4.5) and alkaline (pH = 11) pH was not conducive to enzyme production. Anbu et al. [38] reported pH = 8 as optimal for keratinase activity of the dermatophyte *Trichophyton* sp. HA–2. This was not consistent with the results of our research, which showed that keratinase activity was the highest at the substrate’s initial pH of 4.5 and temperature of 28 °C. It is known, however, that *Trichophyton ajelloi* belongs to the acidophilic fungi, preferring acidic environments [59]. The results of our research also indicated a lower optimum temperature for keratinase activity of the tested *T. ajelloi* strains compared to the results of other authors [20,38]. The optimum temperature for the production of keratinase determined by us was 28 °C. The same fungus species was tested by Kačinová, et al. [20], who showed the highest production of keratinase at slightly higher than the above-mentioned temperature (30 °C); however, at the same time, these authors reported high keratinase production at 37 °C [20]. A similar optimum (35 °C) for keratinase activity, closer to the unidentified *Trichophyton* sp. HA-2 strain, was also recorded by Anbu, et al. [38]. Filamentous fungi are aerobic organisms and their growth is favored by aeration. Kanchana [60] showed that culture aeration by shaking promoted keratinase activity of *Aspergillus* sp., and our research demonstrated that agitation also stimulated the release of active proteases. The main difficulty in enzymatic biodegradation of keratin is the presence of numerous disulfide bridges in the keratin structure [61]. Disulfide reductase is an enzyme involved in breaking down disulfide bridges during biodegradation of native keratin (including feathers), which has previously been detected mainly in bacterial cultures [1,62]. There is little information in the literature regarding the release of these enzymes by keratinolytic fungi. This was indicated by Bhari et al. [63], who reported that both keratinase and disulfide reductase were secreted by bacteria, actinomycetes, and keratinolytic fungi, and their synthesis was induced by the presence of a keratin substrate. Our research showed that disulfide reductase was synthesized by *T. ajelloi* strains and production of this enzyme was favored by the initial pH of the substrate equal to 4.5, 1% feathers and culture temperature of 28 °C. As reported by Rahayu et al. [64], disulfide reductase interacted synergistically with keratinase, which led not only to the reduction of disulfide bridges, but also to the release of proteins containing disulfide bonds. The synergistic activity of these enzymes was also confirmed in the present study. However, this was not a statistically significant effect, as opposed to the interaction of these enzymes in bacterial cells. 

### 4.3. Soluble Proteins and Peptides, Amino Acids and Mineral Biodegradation Products of Chicken Feather Keratin

Some authors [65] reported that proteolysis and sulfitolysis in the process of keratinolysis were simultaneous and complementary phenomena, leading to high nitrogen and high sulfur feather keratin transformation into valuable products of high nutritional value. Bohacz [12] has emphasized that the fungi themselves present in cultivated soils are natural fertilizing agents due to mineral nitrogen and sulfur forms released during biodegradation of native keratin.

Taking into account the release of organic and mineral bonds during keratinolysis of chicken feathers [12,30,31], the current study considered, often omitted by other authors, the effect of different culture conditions and medium composition on the release of high concentrations of soluble proteins and peptides, amino acids, and ammonium and sulfate ions. 

Li [52] determined that the release of soluble proteins, peptides, oligopeptides, and amino acids depended on mutually acting and complementary enzymes. The concentration of these organic products of feather keratin biodegradation by *Trichophyton ajelloi* strains tested in this study increased with culture time, which was consistent with the results of Bohacz [12] in *Chrysosporium articulatum* and *Aphanoascus fulvescens* cultures, and of Maruthi, et al. [66] in *Chrysosporium tropicum* cultures. Soluble proteins and peptides, formed during keratin biodegradation account for the nutritional value of the resulting hydrolysates, which may have applications in animal nutrition [67] and, as reported by Kumari and Kumar [68], also in agriculture due to the presence of amino acids accompanying proteins. Näsholm, et al. [69] believed that amino acids could be absorbed by plants as an N source. Moreover, soluble proteins, peptides and amino acids released in the keratinolysis process constitute fractions of readily hydrolyzing nitrogen, which would undergo further microbial mineralization when introduced into the soil. In the present study, it was shown that, under optimized culture conditions of *Trichophyton ajelloi* fungi, the quantitative and qualitative amino acid composition of the hydrolysates increased significantly compared to amino acid composition under non-optimized conditions in *Arthroderma tuberculatum* and *Arthroderma multifidum* cultures [14]. Considering different cultivation conditions and medium composition, the release of soluble proteins, peptides, and amino acids varies depending on the strain of the microorganism. The effect of 0.09% glucose concentration on the release of soluble proteins from hair by various species of filamentous fungi was investigated by Kaul and Sumbali [70]. These authors recorded in the cultures of *Chrysosporium pannorum* and *Scopulariopsis brevicaulis* more than a 2-fold increase in protein concentration in the variant with glucose. In the study conducted by El-Ghonemy and Ali [57], the release of proteins during keratinolysis by *Aspergillus* sp. DHE7 increased with increasing concentration of feather waste, despite a decrease in keratinase activity. Elevated protein release was also favored by higher (37 °C) temperatures in *Aspergillus* sp. DHE7 cultures, which was also consistent with the results obtained in this study. As demonstrated by Jain, et al. [71], the release of soluble proteins in optimized cultures of the actinobacterium *Streptomyces exfoliatus* CFS 1068 was favored by the initial pH of 8.0, feather concentration of 2% and the presence of glucose, which was also consistent with the results of our own research. Mehta, et al. [72] reported that in the presence of readily available carbon and energy sources such as glucose, sucrose, and mannitol, keratinase secretion in cultures of *Bacillus sonorensis* decreased, and thus the release of amino acids, i.e., the end product of protein degradation. In the cultures of the tested *Trichophyton ajelloi* fungal strains, stimulation of amino acid release, measured by the level of amino and thiol groups, was shown mainly in the presence of glucose. Galarza, et al. [65] reported that keratinases were involved in the release of thiol and amino groups during keratinolysis. Amino acid release, measured by the level of thiol and amino groups in the cultures of *Trichophyton ajelloi* strains, was the most effective when the initial medium pH was higher (6.5 and 8.5), the concentration of chicken feather waste was 2%, and incubation temperature 37 °C; Mehta, et al. [72] made similar observations in bacterial cultures. Additionally, our research showed that the temperature of 28 and 20 °C also stimulated the release of amino acids in *T. ajelloi* cultures growing on feathers. 

Fungi are able to carry out catabolic reactions during keratinolysis, leading to the conversion of cysteine and release of mineral sulfur forms as sulfates and sulfites [48]. Inorganic sulfite released in the process of sulfitolysis plays a role in the reduction of disulfide bridges, contributing to the formation of sulfocysteine, which corresponds to the release of reduced thiols [73]. Many authors in studies on keratinolysis have shown that sulfates are released in the cultures of fungi [12,30,31,48,74], bacteria [62], and actinomycetes. An increase in sulfate content in the medium with increasing culture times was demonstrated by Rajak, et al. [74] in *Chrysosporium indicum, Talaromyces trachyspermus*, *Scopulariopsis brevis*, and *Geotrichum candidum* cultures, by Korniłłowicz–Kowalska [30] in cultures of various fungi from the group of geophilic dermatophytes and *Chrysosporium*, and in recent years by Bohacz [12] and Bohacz, et al. [13,14] in cultures of fungi from the *Chrysosporium* group and the genus *Arthroderma*. In the present study, the first attempt to optimize the cultivation conditions of *Trichophyton ajelloi* showed that the accumulation of high concentrations of sulfate ions in the medium was mainly favored by the temperature of 28 °C, initial pH of 4.5 and feather waste concentration of 2%, while the release of ammonium ions by the presence of xylose, 1 and 2% feather concentration, temperature of 20 and 28 °C, and initial pH of 4.5 and 6.5. 

The release of ammonium ions was accompanied by alkalization of the culture medium, which was consistent with the results of other authors [12,13,14,30,75]. The increase in the concentration of these ions, and thus the gradual increase of medium pH was recorded on day 28, according to the data reported by Bohacz [12] for stationary cultures of *Aphanoascus keratinophilus* and *Chrysosporium articulatum*, and between days 12 and 14 in stationary cultures of *Arthroderma*, i.e., *Arthroderma multifidum*, and *Arthroderma tuberculatum* at initial pH of 6.5, 1% feathers and incubation temperature of 28 °C [14]. *Trichophyton ajelloi* culture conditions optimized in this study showed that the maximum release of ammonium and sulfate ions occurred in the presence of readily available carbon and energy sources, different feather concentrations, culture temperature, initial medium pH, and culture method between days 14 and 21 of solubilization of native keratin by these fungi. We consider species differences in keratinolytic activity to be the reason for these (relatively small) discrepancies between the results of this and previous works [12,13,14] on feather keratinolysis by geophilic dermatophytes and Chrysosporia.

The present study is particularly relevant considering the lack of research in the literature on condition optimization for microorganism cultivation in order to obtain ammonium and sulfate ions—important mineral fertilizer components—as a result of feather waste biodegradation. Particularly important is the availability of bioavailable sulfur in the form of sulfates, because soils in Poland are poor in sulfur, as reported in an earlier study by Bohacz, et al. [26]. Therefore, the intensification of research on fungal or bacterial processing of high-nitrogen and high-sulfur waste into fertilizing preparations is the right step towards obtaining new environmentally friendly biofertilizers. 

## 5. Conclusions

Based on the data obtained during the entire experiment, it has been shown that *Trichophyton ajelloi* has a high ability to decompose hard-to-degrade chicken feather waste. *Trichophyton ajelloi*, due to its keratin waste biodegradation ability, may be considered in the management of keratin-rich waste for its reuse. During the fungal biodegradation of feather keratin, among others, mineral and organic forms of nitrogen and sulfur are released and the resulting keratin hydrolysates can therefore be a valuable biopreparation in terms of fertilization. Optimization of the culture conditions and composition of the culture medium of three strains of *Trichophyton ajelloi* fungus, designated as III, XII, and XIV, in order to obtain the most effective parameters for chicken feather biodegradation, showed that all keratinolysis parameters (keratinase activity, protease, disulfide reductase, feather weight loss and content of organic and mineral forms of nitrogen and sulfur) required different culture parameters and medium composition. Considering the hitherto unoptimized culture conditions and medium composition in terms of obtaining a biopreparation with the highest content of ammonium and sulfate ions, it was demonstrated that all culture conditions favored the generation of sulfate ions, in particular the addition of 2% chicken feathers, temperature of 28 °C and an initial medium pH of 4.5. Concentration of these compounds was recorded at an average of 1.21 mg cm^−3^. The basal control medium, i.e., 1% feathers, pH 4.5, 28 °C (an average of 0.58 mg cm^−3^) was conducive to the release of ammonium ions. Keratin hydrolysate obtained by biodegradation of feather keratin by *Trichophyton ajelloi* strain XII had the highest concentration of mineral forms of nitrogen and sulfur and could be considered as a biopreparation for agricultural application. Research in this area is currently the subject of another publication.

Based on the results of the study, it can be concluded that biological methods of keratin waste management provide products with high fertilizing values through which they contribute to the protection of the environment and public health, as they replace economically unprofitable and environmentally unfriendly chemical methods of their management.

## Figures and Tables

**Table 1:** Size of colony diameter [mm] of *Trichophyton ajelloi* strains.

*T. ajelloi* Strains	Days of Cultivation	Medium	Culture Temperature			
			20 °C	28 °C	37 °C	50 °C
III	7	Sabouraud	27.33 ± 0.47	32.50 ± 1.89	0.00	0.00
		Maltose	16.86 ± 0.37	22.67 ± 1.11	0.00	0.00
	14	Sabouraud	52.17 ± 0.37	55.50 ± 7.46	0.00	0.00
		Maltose	33.83 ± 0.90	43.67 ± 0.75	0.00	0.00
XII	7	Sabouraud	27.33 ± 0.47	34.00 ± 1.15	0.00	0.00
		Maltose	18.00 ± 2.16	24.00 ± 2.89	0.00	0.00
	14	Sabouraud	52.50 ± 1.26	62.67 ± 1.25	0.00	0.00
		Maltose	33.17 ± 2.67	45.00 ± 1.00	0.00	0.00
XIV	7	Sabouraud	22.33 ± 1.25	32.00 ± 1.63	0.00	0.00
		Maltose	14.00 ± 1.41	20.00 ± 0.82	0.00	0.00
	14	Sabouraud	46.83 ± 1.57	64.00 ± 0.82	0.00	0.00
		Maltose	29.33 ± 2.36	41.50 ± 1.80	0.00	0.00

**Table 2:** Hydrolytic capacity of *Trichophyton ajelloi*.

Strain	Enzymatic Index		
	Proteolytic Activity	Lipolytic Activity	Amylolytic Activity
III	1.55 ± 016 ^a^	1.35 ± 0.11 ^a^	0.0
XII	1.59 ± 0.12 ^a^	1.32 ± 0.08 ^a^	0.0
XIV	1.94 ± 0.06 ^b^	1.20 ± 0.08 ^a^	0.0

Explanations: Letters a and b denote means forming homogenous groups, which were determined by one-way ANOVA and HSD–Tukey post-hoc test. If the means are marked with the same letter (e.g., a), they do not differ significantly (at α = 0.05). If the means are given different letters (e.g., a and b, they differ significantly from each other (at α = 0.05).

**Table 3:** Dynamics of pH changes in *Trichophyton ajelloi* culture medium under different culture conditions and medium composition.

*T. ajelloi* Strain	Optimilized Parameters		Days of Analyses				
			7	14	21	28	35
III	Cs	F ^B^	7.81 ± 0.13 ^bc^	8.90 ± 0.04 ^f^	8.77 ± 0.01 ^ef^	8.57 ± 0.02 ^ef^	8.39 ± 0.01 ^def^
		F + G ^A^	6.90 ± 0.03 ^a^	7.41 ± 0.26 ^ab^	8.86 ± 0.04 ^f^	8.82 ± 0.03 ^f^	8.76 ± 0.04 ^ef^
		F + X ^A^	6.93 ± 0.27 ^ab^	7.94 ± 0.37 ^bcd^	8.25 ± 0.15 ^cde^	8.46 ± 0.09 ^def^	8.64 ± 0.03 ^ef^
	Sc [%]	1.0 ^A^	7.81 ± 0.13 ^a^	8.90 ± 0.04 ^def^	8.77 ± 0.01 ^cdef^	8.57 ± 0.02 ^bcd^	8.39 ± 0.01 ^bc^
		1.5 ^C^	8.53 ± 0.04 ^bcd^	9.07 ± 0.01 ^ef^	9.13 ± 0.04 ^f^	9.09 ± 0.01 ^ef^	8.77 ± 0.02 ^cdef^
		2.0 ^B^	8.13 ± 0.36 ^ab^	8.81 ± 0.19 ^cdef^	8.95 ± 0.11 ^def^	8.95 ± 0.09 ^def^	8.67 ± 0.08 ^cde^
	pH	4.5 ^A^	7.81 ± 0.13 ^a^	8.90 ± 0.04 ^fg^	8.77 ± 0.01 ^ef^	8.57 ± 0.02 ^bcd^	8.39 ± 0.01 ^b^
		6.5 ^B^	8.94 ± 0.03 ^fg^	9.24 ± 0.02 ^hi^	9.22 ± 0.04 ^hi^	8.42 ± 0.08 ^bc^	8.70 ± 0.05 ^de^
		8.5 ^C^	9.08 ± 0.03 ^gh^	9.32 ± 0.02 ^i^	9.28 ± 0.01 ^i^	8.61 ± 0.01 ^cde^	9.01 ± 0.09 ^g^
	T [°C]	20 ^C^	7.76 ± 0.11 ^c^	8.93 ± 0.02 ^fg^	9.08 ± 0.03 ^g^	9.10 ± 0.02 ^g^	8.90 ± 0.02 ^fg^
		28 ^B^	7.81 ± 0.13 ^c^	8.90 ± 0.04 ^fg^	8.77 ± 0.01 ^ef^	8.57 ± 0.02 ^de^	8.39 ± 0.01 ^d^
		37 ^A^	6.39 ± 0.08 ^a^	6.48 ± 0.08 ^a^	6.72 ± 0.07 ^b^	6.88 ± 0.06 ^b^	6.92 ± 0.05 ^b^
	Cm	STA ^A^	7.81 ± 0.13 ^bc^	8.90 ± 0.04 ^a^	8.77 ± 0.01 ^a^	8.57 ± 0.02 ^a^	8.39 ± 0.01 ^ab^
		AG ^A^	7.68 ± 0.45 ^c^	8.40 ± 0.27 ^ab^	8.76 ± 0.12 ^a^	8.67 ± 0.10 ^a^	8.65 ± 0.12 ^a^
XII	Cs	F ^C^	8.55 ± 0.02 ^bcde^	8.79 ± 0.01 ^def^	8.66 ± 0.13 ^cdef^	8.71 ± 0.01 ^cdef^	8.51 ± 0.02 ^bcd^
		F + G ^B^	7.10 ± 0.12 ^a^	8.36 ± 0.13 ^bc^	8.93 ± 0.01 ^f^	8.92 ± 0.03 ^f^	8.87 ± 0.03 ^ef^
		F + X ^A^	7.00 ± 0.24 ^a^	8.21 ± 0.12 ^b^	8.49 ± 0.08 ^bcd^	8.53 ± 0.07 ^bcde^	8.64 ± 0.08 ^cdef^
	Sc [%]	1.0A ^B^	8.55 ± 0.02 ^bc^	8.79 ± 0.01 ^bc^	8.66 ± 0.13 ^bc^	8.71 ± 0.01 ^bc^	8.51 ± 0.02 ^bc^
		1.5 ^A^	8.98 ± 0.49 ^e^	9.21 ± 0.28 ^fg^	9.17 ± 0.19 ^fg^	8.48 ± 0.12 ^a^	8.68 ± 0.06 ^cd^
		2.0 ^B^	9.06 ± 0.21 ^ef^	9.32 ± 0.16 ^g^	9.27 ± 0.06 ^g^	8.54 ± 0.03 ^abc^	8.99 ± 0.04 ^e^
	pH	4.5 ^A^	8.55 ± 0.02 ^abc^	8.79 ± 0.01 ^d^	8.66 ± 0.13 ^bcd^	8.71 ± 0.01 ^cd^	8.51 ± 0.02 ^ab^
		6.5 ^B^	7.74 ± 0.06 ^a^	8.67 ± 0.01 ^bc^	8.86 ± 0.03 ^bc^	8.86 ± 0.01 ^bc^	8.63 ± 0.04 ^bc^
		8.5 ^C^	8.36 ± 0.02 ^ab^	8.68 ± 0.02 ^bc^	9.05 ± 0.01 ^c^	9.08 ± 0.03 ^c^	8.72 ± 0.06 ^bc^
	T [°C]	20 ^B^	6.99 ± 0.19 ^b^	8.57 ± 0.18 ^c^	8.88 ± 0.12 ^c^	8.92 ± 0.10 ^c^	8.75 ± 0.11 ^c^
		28 ^C^	8.55 ± 0.02 ^c^	8.79 ± 0.01 ^c^	8.66 ± 0.13 ^c^	8.71 ± 0.01 ^c^	8.51 ± 0.02 ^c^
		37 ^A^	6.35 ± 0.10 ^a^	6.48 ± 0.08 ^a^	6.65 ± 0.16 ^ab^	6.67 ± 0.12 ^ab^	6.66 ± 0.21 ^ab^
	Cm	STA ^A^	8.55 ± 0.02 ^abc^	8.79 ± 0.01 ^ab^	8.66 ± 0.13 ^abc^	8.71 ± 0.01 ^ab^	8.51 ± 0.02 ^ac^
		AG ^A^	8.23 ± 0.11 ^c^	8.56 ± 0.35 ^abc^	8.98 ± 0.06 ^b^	8.72 ± 0.11 ^ab^	8.60 ± 0.07 ^abc^
XIV	Cs	F ^C^	8.44 ± 0.06 ^de^	8.78 ± 0.05 ^e^	8.58 ± 0.05 ^de^	8.82 ± 0.04 ^e^	8.51 ± 0.04 ^de^
		F + G ^B^	7.03 ± 0.17 ^ab^	7.62 ± 0.05 ^bc^	8.79 ± 0.10 ^e^	8.84 ± 0.06 ^e^	8.88 ± 0.07 ^e^
		F + X ^A^	6.70 ± 0.23 ^a^	7.57 ± 0.37 ^bc^	8.01 ± 0.42 ^cd^	8.59 ± 0.04 ^de^	8.61 ± 0.03 ^de^
	Sc [%]	1.0 ^A^	8.44 ± 0.06 ^cd^	8.78 ± 0.05 ^defg^	8.58 ± 0.05 ^cdef^	8.82 ± 0.04 ^defg^	8.51 ± 0.04 ^cdef^
		1.5 ^B^	7.24 ± 0.16 ^a^	8.35 ± 0.13 ^c^	8.70 ± 0.10 ^cdefg^	8.75 ± 0.10 ^defg^	8.56 ± 0.10 ^cdef^
		2.0 ^A^	7.84 ± 0.28 ^b^	8.99 ± 0.07 ^g^	8.90 ± 0.08 ^fg^	8.87 ± 0.04 ^efg^	8.49 ± 0.05 ^cde^
	pH	4.5 ^A^	8.44 ± 0.06 ^a^	8.78 ± 0.05 ^def^	8.58 ± 0.05 ^abcd^	8.82 ± 0.04 ^ef^	8.51 ± 0.04 ^abc^
		6.5 ^B^	8.92 ± 0.03 ^fg^	9.18 ± 0.04 ^h^	9.17 ± 0.05 ^h^	8.45 ± 0.05 ^ab^	8.67 ± 0.02 ^bcde^
		8.5 ^C^	9.10 ± 0.12 ^gh^	9.28 ± 0.02 ^h^	9.26 ± 0.03 ^h^	8.68 ± 0.12 ^cde^	9.07 ± 0.10 ^gh^
	T [°C]	20 ^C^	8.08 ± 0.05 ^b^	8.95 ± 0.02 ^e^	9.06 ± 0.04 ^e^	9.08 ± 0.03 ^e^	8.85 ± 0.03 ^de^
		28 ^B^	8.44 ± 0.06 ^c^	8.78 ± 0.05 ^cde^	8.58 ± 0.05 ^cd^	8.82 ± 0.04 ^de^	8.51 ± 0.04 ^cd^
		37 ^A^	6.05 ± 0.10 ^a^	6.09 ± 0.12 ^a^	6.19 ± 0.12 ^a^	6.20 ± 0.13 ^a^	6.16 ± 0.25 ^a^
	Cm	STA ^A^	8.44 ± 0.06 ^b^	8.78 ± 0.05 ^cd^	8.58 ± 0.05 ^ab^	8.82 ± 0.04 ^cd^	8.51 ± 0.04 ^ab^
		AG ^A^	8.19 ± 0.04 ^e^	8.70 ± 0.05 ^ac^	8.93 ± 0.02 ^d^	8.65 ± 0.04 ^ac^	8.53 ± 0.11 ^ab^

Explanations: Letters, i.e., lowercase (a, b, c) or capital (A, B, C) denote the means that form homogenous groups (HSD–Tukey post-hoc test for multivariate ANOVA). When two compared means were assigned the same letter (e.g., a or A), that means did not differ significantly (at α = 0.05); if means are marked with different letters (e.g., a and b, or A and B, they differ significantly from each other (at α = 0.05); Cs—carbon sources, Sc—substrate concentration, T—culture temperature, Cm—culture method; F—cultures with feathers as a sole carbon source; F + G—cultures with feathers and glucose addition; F + X—cultures with feathers and xylose addition; STA—stationary cultures; AG —agitated cultures.

**Table 4:** Degree of keratin substrate degradation (%) by *Trichophyton ajelloi* fungal strains under various culture conditions and medium composition.

Optimized Parameters		*T. ajelloi* Strains		
		III ^A^	XII ^A^	XIV ^A^
Carbon sources	F ^C^	37.33 ± 4.64 ^d^	36.90 ± 0.62 ^d^	36.57 ± 1.37 ^d^
	F + G ^A^	5.10 ± 0.29 ^a^	5.03 ± 1.46 ^a^	8.97 ± 1.32 ^ab^
	F + X ^B^	15.50 ± 0.94 ^bc^	14.77 ± 0.45 ^bc^	16.63 ± 2.33 ^c^
Substrate concentration	1.0% ^A^	37.33 ± 4.64 ^a^	36.90 ± 0.62 ^a^	36.57 ± 1.37 ^a^
	1.5% ^A^	41.43 ± 3.95 ^ac^	38.30 ± 7.22 ^a^	35.47 ± 4.29 ^a^
	2.0% ^B^	57.30 ± 5.51 ^b^	59.07 ± 3.54 ^b^	56.00 ± 5.53 ^bc^
Initial pH of culture medium	4.5 ^C^	37.33 ± 4.64 ^c^	36.90 ± 0.62 ^c^	36.57 ± 1.37 ^bd^
	6.5 ^B^	32.93 ± 0.29 ^bcd^	31.47 ± 0.78 ^abcd^	30.77 ± 1.03 ^abd^
	8.5 ^A^	27.33 ± 0.42 ^ab^	26.90 ± 0.14 ^a^	27.00 ± 0.79 ^ab^
Culture temperature	20 °C ^B^	31.67 ± 1.28 ^ab^	29.77 ± 2.79 ^a^	34.17 ± 0.82 ^ab^
	28 °C ^C^	37.33 ± 4.64 ^b^	36.90 ± 0.62 ^ab^	36.57 ± 1.37 ^ab^
	37 °C ^A^	17.33 ± 0.82 ^c^	19.70 ± 1.04 ^c^	20.97 ± 2.26 ^c^
Culture type	STA ^B^	37.33 ± 4.64 ^b^	36.90 ± 0.62 ^b^	36.57 ± 1.37 ^b^
	AG ^A^	21.77 ± 1.64 ^a^	20.33 ± 1.85 ^a^	16.00 ± 0.29 ^a^

Explanations: Letters, i.e., lowercase (a, b, c) or capital (A, B, C) denote the means that form homogenous groups (HSD–Tukey post-hoc test for multivariate ANOVA). When two compared means were assigned the same letter (e.g., a or A), that means did not differ significantly (at α = 0.05); if means are marked with different letters (e.g., a and b, or A and B, they differ significantly from each other (at α = 0.05); F + G—cultures with feathers and glucose addition; F + X—cultures with feathers and xylose addition; STA—stationary cultures; AG —agitated cultures.

**Table 5:** Amino acid composition [µg g^–1^] of 18–day culture fluids of *Trichophyton ajelloi* strain III; feathers + glucose (F + G); feathers + xylose (F + X); agitated culture (AG).

Amino Acids	Optimized Parameters					
	20 °C	37 °C	F + G	F + X	AG	Total
Asp	33.30	65.30	39.00	50.10	52.10	239.80
Thr	8.30	31.00	11.60	19.90	20.40	91.20
Ser	25.60	35.80	25.20	31.10	19.80	137.50
Glu	20.60	93.10	31.00	69.60	49.20	263.50
Pro	13.10	18.40	128.00	0.00	12.80	172.30
Gly	18.50	42.30	14.30	30.60	22.60	128.30
Ala	6.00	42.20	10.70	18.40	27.00	104.30
Cyst. acid	63.60	11.00	48.30	32.00	37.80	192.70
Val	2.90	34.10	10.10	21.70	15.40	84.20
Sulf met	4.58	9.45	5.73	9.69	7.04	36.49
Ile	2.20	28.60	8.00	16.00	29.90	84.70
Leu	4.30	46.00	6.80	26.00	18.70	101.80
Tyr	0.00	24.80	5.20	13.50	8.20	51.70
Phe	4.40	23.70	0.00	10.20	10.40	48.70
His	8.00	6.50	5.40	3.80	10.90	34.60
Lys	10.40	44.10	11.90	21.50	28.60	116.50
Arg	5.80	35.30	5.80	14.90	7.80	69.60
Trp	132.00	139.00	87.00	244.00	129.00	731.00
Total	363.58	730.65	454.03	632.99	507.64	

**Table 6:** Differences in the ability of *Trichophyton ajelloi* strains to biodegrade chicken feather waste.

Parameters	*Trichophyton ajelloi* Strains		
	III	XII	XIV
Protease [µg tyrosine cm^−3^]	59.14 ± 5.02 ^b^	56.41 ± 4.73 ^a^	54.78 ± 5.60 ^a^
Keratinase [KU cm^−3^]	58.99 ± 4.08 ^b^	53.41 ± 3.42 ^a^	53.79 ± 3.85 ^a^
Disulfide reductase [U cm^−3^]	0.04 ± 0.01 ^a^	0.05 ± 0.01 ^c^	0.04 ± 0.01 ^b^
Proteins and peptides [µg proteins cm^−3^]	559.10 ± 21.58 ^a^	551.86 ± 18.61 ^a^	534.88 ± 23.57 ^b^
Amino groups [µg N–NH_2_ cm^−3^]	18.72 ± 0.82 ^a^	18.65 ± 0.75 ^a^	18.19 ± 0.60 ^b^
Sulfhydryl groups [µg −SH cm^−3^]	5.57 ± 0.58 ^b^	7.02 ± 0.75 ^a^	6.80 ± 0.73 ^a^
Ammonium ions [NH^4+^ cm^−3^]	472.47 ± 24.80 ^a^	475.80 ± 22.31 ^a^	441.80 ± 21.06 ^b^
Sulfate ions [mg SO_4_^2–^ cm^−3^]	0.76 ± 0.03 ^b^	0.78 ± 0.03 ^c^	0.71 ± 0.03 ^a^
pH [−log_10_ [H^+^]]	8.41 ± 0.09 ^a^	8.41 ± 0.10 ^a^	8.32 ± 0.09 ^b^

Explanations: Letters, i.e., a, b, and c denote means forming homogenous groups (HSD–Tukey post–hoc test for multivariate ANOVA). When two compared means were assigned the same letter (e.g., a), they did not differ significantly (at α = 0.05); if means were given different letters (e.g., a and b or A and B, then they differed significantly from each other (α = 0.05).

## Data Availability

All data is contained within the article.

## Supplementary Materials

The following supporting information can be downloaded at: https://www.mdpi.com/article/10.3390/ijerph191710858/s1, Table S1: Dynamics of changes in enzymatic activity and secretion of organic and mineral products of chicken feather keratinolysis by *Trichophyton ajelloi* strains with the addition of various carbon sources to the culture medium; Table S2: Dynamics of changes in enzymatic activity and secretion of organic and mineral products of chicken feather keratinolysis by *Trichophyton ajelloi* strains at various concentrations (1.0, 1.5 and 2.0%) of feather waste; Table S3: Dynamics of changes in enzymatic activity and secretion of organic and mineral products of chicken feather keratinolysis by *Trichophyton ajelloi* strains at different initial pH (4.5, 6.5, 8.5) of the culture medium, Table S4: Dynamics of changes in enzymatic activity and secretion of organic and mineral products of chicken feather keratinolysis by *Trichophyton ajelloi* strains fungi in various culture temperature variants (20, 28, and 37 °C); Table S5: Dynamics of changes in enzymatic activity and secretion of organic and mineral products of chicken feather keratinolysis of by *Trichophyton ajelloi* strains with different culture methods.
