# Supplementary material for: Optimization of Conditions for Feather Waste Biodegradation by Geophilic Trichophyton ajelloi Fungal Strains towards Further Agricultural Use
Source: Int J Environ Res Public Health. 2022 Aug 31;19(17):10858. doi: 10.3390/ijerph191710858 (PMC9518355; doi:10.3390/ijerph191710858)
Supplement: Supplementary file 1 [file ijerph-19-10858-s001.zip › ijerph-1865971-supplementary.pdf]

Supplementary material for International Journal of Environmental Research and Public Health

Optimization of conditions for feather waste biodegradation by geophilic *Trichophyton ajelloi* fungal strains towards further agricultural use

Michał Możejko[1], Justyna Bohacz [1]

[1] Department of Environmental Microbiology, Faculty of Agrobioengineering, University of Life Sciences in Lublin, Leszczyńskiego 7 Street, 20–069 Lublin, Poland

Corresponding author:
Justyna Bohacz, e-mail address: justyna.bohacz@up.lublin.pl
phone: +48 81 524 81 04; fax: +48 81 524 81 06

Table S1. Dynamics of changes in enzymatic activity and the secretion of organic and mineral products of chicken feathers keratinolysis by *Trichophyton ajelloi* strains with the addition of various carbon sources to the culture medium.

Days	7	14	21	28	35	Mean
**Protease [μg tyrosine cm^{-3}]**						
F (III)	48.15 ± 3.90^{a}	58.87 ± 3.26^{b}	40.24 ± 0.55^{a}	59.02 ± 3.99^{b}	70.56 ± 1.27^{c}	55.37 ± 2.59
F (XII)	44.82 ± 1.09^{ac}	47.56 ± 2.08^{a}	37.57 ± 1.64^{b}	40.01 ± 1.27^{bc}	49.41 ± 2.16^{a}	43.88 ± 1.65
F (XIV)	49.04 ± 2.54^{c}	38.09 ± 3.02^{ab}	32.84 ± 0.79^{a}	33.14 ± 3.26^{a}	42.16 ± 1.57^{bc}	39.05 ± 2.24
F+G (III)	16.86 ± 2.20^{c}	82.25 ± 11.05^{ab}	92.08 ± 5.28^{b}	68.05 ± 3.71^{a}	73.74 ± 2.73^{ab}	66.60 ± 4.99
F+G (XII)	21.75 ± 1.48^{c}	99.26 ± 3.73^{a}	98.08 ± 8.01^{a}	83.21 ± 1.92^{b}	92.75 ± 3.18^{ab}	79.01 ± 3.66
F+G (XIV)	31.73 ± 7.42^{a}	107.54 ± 16.58^{b}	93.86 ± 10.27^{b}	59.39 ± 5.50^{a}	59.91 ± 4.45^{a}	70.49 ± 8.84
F+X (III)	16.79 ± 2.24^{c}	41.94 ± 6.71^{ab}	42.16 ± 8.18^{ab}	36.46 ± 0.82^{a}	56.95 ± 4.96^{b}	38.86 ± 4.58
F+X (XII)	13.68 ± 1.45^{b}	38.39 ± 3.99^{a}	34.91 ± 2.73^{a}	45.27 ± 9.55^{a}	74.70 ± 3.63^{c}	41.39 ± 4.27
F+X (XIV)	15.24 ± 1.09^{b}	38.09 ± 4.12^{ab}	45.86 ± 17.49^{ab}	50.81 ± 8.34^{a}	67.38 ± 12.89^{a}	43.48 ± 8.79
**Keratinase [KU cm^{-3}]**						
F (III)	54.77 ± 1.68^{b}	79.57 ± 1.75^{a}	81.03 ± 1.54^{a}	80.73 ± 2.78^{a}	93.70 ± 1.69^{c}	77.96 ± 1.89
F (XII)	40.23 ± 2.05^{a}	38.53 ± 1.55^{a}	48.40 ± 2.27^{b}	44.30 ± 2.60^{ab}	56.97 ± 1.62^{c}	45.69 ± 2.02
F (XIV)	39.67 ± 2.48^{a}	42.87 ± 1.02^{ab}	49.13 ± 2.52^{bc}	50.27 ± 1.62^{c}	56.80 ± 1.39^{d}	47.75 ± 1.81
F+G (III)	49.90 ± 3.19^{c}	79.23 ± 5.39^{d}	111.43 ± 7.84^{ab}	110.63 ± 6.37^{a}	129.73 ± 4.34^{b}	96.19 ± 5.43
F+G (XII)	37.10 ± 6.08^{b}	57.80 ± 6.37^{c}	86.00 ± 7.08^{a}	78.13 ± 3.33^{a}	109.71 ± 1.88^{d}	73.75 ± 4.95
F+G (XIV)	38.80 ± 4.46^{b}	45.87 ± 6.18^{b}	95.23 ± 7.11^{a}	103.93 ± 2.95^{a}	108.60 ± 3.41^{a}	78.49 ± 4.82
F+X (III)	51.30 ± 3.12^{a}	42.97 ± 3.61^{a}	68.23 ± 6.59^{b}	55.40 ± 2.91^{ab}	92.13 ± 5.49^{c}	62.01 ± 4.35
F+X (XII)	42.23 ± 1.65^{a}	41.40 ± 1.82^{a}	54.93 ± 5.83^{b}	51.97 ± 1.54^{ab}	92.33 ± 4.32^{c}	56.57 ± 3.03
F+X (XIV)	37.57 ± 5.72^{b}	65.87 ± 8.58^{a}	62.63 ± 3.87^{a}	54.80 ± 4.03^{ab}	97.70 ± 6.65^{c}	63.71 ± 5.77
**Disulfide reductase [U cm^{-3}]**						
F (III)	0.010 ± 0.001^{a}	0.013 ± 0.005^{a}	0.013 ± 0.005^{a}	0.019 ± 0.008^{ab}	0.032 ± 0.005^{b}	0.017 ± 0.004
F (XII)	0.194 ± 0.008^{ab}	0.188 ± 0.012^{a}	0.223 ± 0.008^{bc}	0.207 ± 0.012^{ab}	0.256 ± 0.012^{c}	0.214 ± 0.010
F (XIV)	0.013 ± 0.005^{a}	0.036 ± 0.009^{b}	0.019 ± 0.008^{ab}	0.026 ± 0.005^{ab}	0.084 ± 0.005^{c}	0.036 ± 0.006
F+G (III)	0.052 ± 0.005^{c}	0.013 ± 0.005^{a}	0.013 ± 0.005^{a}	0.032 ± 0.005^{b}	0.026 ± 0.005^{ab}	0.027 ± 0.005
F+G (XII)	0.032 ± 0.005^{ab}	0.013 ± 0.005^{a}	0.019 ± 0.008^{ab}	0.016 ± 0.005^{a}	0.039 ± 0.008^{b}	0.024 ± 0.006
F+G (XIV)	0.068 ± 0.008^{b}	0.091 ± 0.012^{b}	0.010 ± 0.001^{c}	0.036 ± 0.005^{a}	0.036 ± 0.005^{a}	0.048 ± 0.006
F+X (III)	0.207 ± 0.012^{c}	0.032 ± 0.012^{a}	0.142 ± 0.017^{b}	0.013 ± 0.005^{a}	0.049 ± 0.016^{a}	0.089 ± 0.012
F+X (XII)	0.136 ± 0.008^{b}	0.010 ± 0.001^{a}	0.013 ± 0.005^{a}	0.013 ± 0.005^{a}	0.010 ± 0.001^{a}	0.036 ± 0.003
F+X (XIV)	0.029 ± 0.008^{a}	0.013 ± 0.005^{a}	0.058 ± 0.008^{b}	0.026 ± 0.005^{a}	0.013 ± 0.005^{a}	0.028 ± 0.006
**Proteins and peptides [μg of proteins cm^{-3}]**						
F (III)	297.42 ± 5.44^{b}	473.18 ± 7.52^{a}	473.37 ± 6.33^{a}	509.37 ± 7.35^{c}	545.95 ± 19.68^{d}	459.86 ± 9.26
F (XII)	341.12 ± 20.62^{b}	421.78 ± 14.01^{a}	387.32 ± 9.27^{ab}	394.06 ± 14.68^{a}	430.25 ± 15.16^{a}	394.91 ± 14.75
F (XIV)	308.40 ± 7.41^{d}	394.06 ± 1.91^{bc}	365.19 ± 2.37^{a}	378.85 ± 6.48^{ab}	413.12 ± 10.90^{c}	371.92 ± 5.81
F+G (III)	756.55 ± 13.59^{a}	498.78 ± 46.63^{b}	720.17 ± 25.67^{a}	692.25 ± 19.61^{a}	716.51 ± 23.49^{a}	676.85 ± 25.80
F+G (XII)	775.80 ± 7.22^{e}	471.64 ± 12.43^{a}	619.49 ± 5.70^{b}	677.43 ± 2.63^{c}	724.79 ± 8.66^{d}	653.83 ± 7.33
F+G (XIV)	790.82 ± 11.59^{d}	435.84 ± 17.91^{c}	624.68 ± 31.34^{a}	665.88 ± 25.78^{ab}	705.92 ± 23.98^{b}	644.63 ± 22.12
F+X (III)	934.62 ± 31.39^{b}	1227.62 ± 39.60^{a}	1299.81 ± 17.49^{a}	1217.22 ± 29.43^{a}	1283.63 ± 22.80^{a}	1192.58 ± 28.14
F+X (XII)	933.46 ± 26.93^{c}	1253.80 ± 13.59^{ab}	1213.95 ± 39.76^{ab}	1189.11 ± 25.39^{a}	1288.83 ± 33.92^{b}	1175.83 ± 27.92
F+X (XIV)	932.12 ± 26.29^{b}	968.89 ± 46.82^{b}	1092.09 ± 39.24^{a}	1118.46 ± 33.59^{a}	1192.96 ± 32.80^{a}	1060.90 ± 35.75
**Amino groups [μg N-NH$_2$ cm^{-3}]**						
F (III)	5.78 ± 0.43^{a}	10.62 ± 0.23^{b}	10.34 ± 0.30^{b}	6.16 ± 0.33^{a}	6.61 ± 0.19^{a}	7.90 ± 0.30
F (XII)	7.02 ± 0.16^{b}	10.89 ± 0.15^{c}	11.98 ± 0.31^{a}	12.57 ± 0.34^{a}	14.46 ± 0.33^{d}	11.38 ± 0.26
F (XIV)	5.37 ± 0.31^{b}	8.32 ± 0.24^{a}	9.15 ± 0.28^{a}	7.32 ± 0.17^{c}	8.42 ± 0.32^{a}	7.72 ± 0.26
F+G (III)	2.07 ± 0.01^{b}	11.09 ± 0.24^{c}	21.02 ± 0.60^{d}	24.60 ± 0.99^{a}	24.55 ± 0.77^{a}	16.67 ± 0.52
F+G (XII)	2.14 ± 0.52^{b}	14.24 ± 1.29^{c}	18.34 ± 1.16^{d}	29.66 ± 0.37^{a}	29.81 ± 0.95^{a}	18.84 ± 0.86
F+G (XIV)	2.54 ± 0.05^{b}	10.10 ± 0.37^{c}	22.23 ± 1.73^{d}	28.64 ± 0.91^{a}	31.66 ± 1.35^{a}	19.03 ± 0.88
F+X (III)	2.47 ± 0.12^{c}	17.89 ± 1.02^{a}	20.72 ± 1.44^{a}	26.16 ± 1.19^{b}	29.85 ± 1.90^{b}	19.42 ± 1.13
F+X (XII)	2.99 ± 0.07^{c}	18.83 ± 0.59^{a}	21.10 ± 1.02^{ab}	24.07 ± 0.83^{b}	27.51 ± 1.61^{d}	18.90 ± 0.82

F+X (XIV)	2.86 ± 0.34^{a}	14.08 ± 0.48^{b}	20.36 ± 0.63^{c}	27.06 ± 1.10^{d}	29.76 ± 0.74^{e}	18.82 ± 0.66
**Thiol groups [μg -SH cm^{-3}]**						
F (III)	2.35 ± 0.15^{a}	2.99 ± 0.15^{ab}	6.72 ± 0.47^{d}	4.69 ± 0.15^{c}	3.95 ± 0.53^{bc}	4.14 ± 0.29
F (XII)	5.28 ± 0.13^{ab}	7.14 ± 0.87^{c}	3.73 ± 0.40^{a}	4.37 ± 0.33^{a}	6.13 ± 0.42^{bc}	5.33 ± 0.43
F (XIV)	4.80 ± 0.23^{a}	5.33 ± 0.33^{a}	4.53 ± 0.87^{a}	4.69 ± 0.53^{a}	4.74 ± 0.96^{a}	4.82 ± 0.58
F+G (III)	0.43 ± 0.27^{c}	4.96 ± 1.12^{b}	8.80 ± 1.51^{a}	8.48 ± 1.26^{ab}	10.13 ± 0.80^{a}	6.56 ± 0.99
F+G (XII)	0.21 ± 0.08^{b}	4.58 ± 0.98^{c}	8.80 ± 1.71^{a}	8.58 ± 0.89^{a}	10.34 ± 0.98^{a}	6.50 ± 0.93
F+G (XIV)	1.44 ± 0.23^{a}	0.85 ± 0.20^{a}	5.54 ± 0.89^{b}	7.36 ± 1.45^{b}	11.78 ± 1.28^{c}	5.40 ± 0.81
F+X (III)	6.40 ± 0.91^{c}	2.67 ± 0.49^{ab}	3.52 ± 0.82^{b}	1.33 ± 0.27^{a}	2.13 ± 0.15^{ab}	3.21 ± 0.53
F+X (XII)	7.25 ± 1.32^{b}	4.74 ± 0.59^{ab}	3.52 ± 0.94^{a}	4.27 ± 0.72^{ab}	4.90 ± 0.80^{ab}	4.94 ± 0.87
F+X (XIV)	3.89 ± 0.64^{a}	2.67 ± 0.54^{a}	4.16 ± 0.45^{a}	4.43 ± 0.40^{a}	7.62 ± 1.57^{b}	4.55 ± 0.72
**Sulfate ions [mg SO_4^{2-} cm^{-3}]**						
F (III)	0.32 ± 0.03^{a}	0.95 ± 0.02^{d}	1.15 ± 0.02^{e}	0.55 ± 0.03^{b}	0.66 ± 0.03^{c}	0.73 ± 0.02
F (XII)	0.59 ± 0.04^{a}	0.96 ± 0.04^{c}	0.57 ± 0.03^{a}	0.17 ± 0.01^{b}	0.53 ± 0.02^{a}	0.56 ± 0.03
F (XIV)	0.34 ± 0.03^{a}	1.00 ± 0.06^{b}	0.91 ± 0.01^{b}	0.31 ± 0.10^{a}	0.60 ± 0.02^{c}	0.63 ± 0.04
F+G (III)	0.35 ± 0.02^{b}	0.46 ± 0.02^{c}	0.98 ± 0.02^{d}	0.91 ± 0.03^{a}	0.88 ± 0.02^{a}	0.71 ± 0.02
F+G (XII)	0.34 ± 0.02^{b}	0.71 ± 0.03^{c}	0.94 ± 0.04^{a}	0.92 ± 0.04^{a}	0.87 ± 0.02^{a}	0.76± 0.03
F+G (XIV)	0.48 ± 0.02^{b}	0.50 ± 0.03^{b}	1.02 ± 0.06^{a}	1.03 ± 0.01^{a}	0.95 ± 0.02^{a}	0.80 ± 0.03
F+X (III)	0.22 ± 0.01^{b}	0.49 ± 0.04^{c}	0.74 ± 0.06^{a}	0.65 ± 0.03^{a}	1.02 ± 0.02^{d}	0.62 ± 0.03
F+X (XII)	0.43 ± 0.02^{a}	0.53 ± 0.01^{a}	0.81 ± 0.01^{b}	0.77 ± 0.06^{b}	1.02 ± 0.04^{c}	0.71 ± 0.03
F+X (XIV)	0.26 ± 0.02^{b}	0.42 ± 0.04^{a}	0.48 ± 0.03^{a}	0.82 ± 0.04^{c}	0.94 ± 0.03^{d}	0.58 ± 0.03
**Ammonium ions [μg NH^{4+} cm^{-3}]**						
F (III)	365.53 ± 7.71^{c}	637.65 ± 9.38^{a}	620.27 ± 23.04^{a}	558.61 ± 7.19^{b}	598.95 ± 12.50^{ab}	556.20 ± 11.96
F (XII)	525.24 ± 7.75^{b}	687.03 ± 8.92^{a}	677.99 ± 5.42^{a}	555.37 ± 9.20^{c}	593.61 ± 10.50^{d}	607.85 ± 8.36
F (XIV)	460.33 ± 14.23^{b}	646.23 ± 2.29^{a}	668.71 ± 11.49^{a}	531.49 ± 8.98^{c}	578.08 ± 6.02^{d}	576.97 ± 8.60
F+G (III)	258.68 ± 6.55^{b}	286.49 ± 10.52^{b}	549.81 ± 3.13^{a}	562.55 ± 23.61^{a}	548.42 ± 11.70^{a}	441.19 ± 11.10
F+G (XII)	307.82 ± 27.87^{b}	403.08 ± 34.79^{c}	569.04 ± 13.06^{a}	583.18 ± 2.36^{a}	513.41 ± 22.46^{a}	475.31 ± 20.11
F+G (XIV)	308.05 ± 22.43^{b}	287.88 ± 37.37^{b}	487.69 ± 35.84^{a}	546.33 ± 36.16^{a}	569.04 ± 21.38^{a}	439.80 ± 30.64
F+X (III)	309.67 ± 19.88^{b}	499.04 ± 26.97^{c}	712.06 ± 53.76^{a}	677.52 ± 37.06^{a}	761.20 ± 24.59^{a}	591.90 ± 32.45
F+X (XII)	330.53 ± 37.86^{d}	528.94 ± 30.48^{a}	623.98 ± 27.28^{ab}	708.81 ± 16.43^{bc}	775.57 ± 45.92^{c}	593.57 ± 31.59
F+X (XIV)	303.64 ± 25.92^{c}	486.30 ± 32.72^{b}	621.89 ± 41.79^{ab}	689.11 ± 48.10^{a}	707.89 ± 57.38^{a}	561.77 ± 41.18

Explanations: Letters a, b, and c denote means forming homogenous groups, which were determined by one–way ANOVA and HSD–Tukey post–hoc test. If the means are marked with the same letter (e.g. a), they do not differ significantly (at $\alpha = 0.05$). If the means are given different letters (e.g.: a and b, they differ significantly from each other (at $\alpha = 0.05$).

Table S2. Dynamics of changes in enzymatic activity and the secretion of organic and mineral products of chicken feathers keratinolysis by *Trichophyton ajelloi* strains at various concentrations (1.0, 1.5 and 2.0%) of feather waste.

Days	7	14	21	28	35	Mean
**Protease [µg tyrosine cm^{-3}]**						
1.0% (III)	48.15 ± 3.90^{a}	58.87 ± 3.26^{b}	40.24 ± 0.55^{a}	59.02 ± 3.99^{b}	70.56 ± 1.27^{c}	55.37 ± 2.59
1.0% (XII)	44.82 ± 1.09^{ac}	47.56 ± 2.08^{a}	37.57 ± 1.64^{b}	40.01 ± 1.27^{bc}	49.41 ± 2.16^{a}	43.88 ± 1.65
1.0% (XIV)	49.04 ± 2.54^{c}	38.09 ± 3.02^{ab}	32.84 ± 0.79^{a}	33.14 ± 3.26^{a}	42.16 ± 1.57^{bc}	39.05 ± 2.24
1.5% (III)	25.15 ± 3.02^{b}	39.87 ± 4.01^{a}	39.94 ± 4.26^{a}	34.54 ± 2.21^{ab}	37.43 ± 2.60^{a}	35.38 ± 3.22
1.5% (XII)	40.38 ± 2.38^{ab}	51.70 ± 4.08^{b}	46.38 ± 2.98^{ab}	38.17 ± 5.16^{a}	37.20 ± 1.48^{a}	42.77 ± 3.22
1.5% (XIV)	36.17 ± 1.96^{a}	50.89 ± 9.60^{a}	60.06 ± 17.68^{a}	66.35 ± 9.67^{a}	71.30 ± 9.81^{a}	56.95 ± 9.74
2.0% (III)	36.76 ± 1.09^{b}	62.50 ± 1.37^{a}	64.72 ± 3.68^{a}	56.66 ± 0.52^{a}	57.54 ± 5.49^{a}	55.64 ± 2.43
2.0% (XII)	33.28 ± 2.44^{b}	58.65 ± 9.01^{a}	57.47 ± 3.57^{a}	51.11 ± 3.17^{ab}	56.29 ± 7.08^{a}	51.36 ± 5.05
2.0% (XIV)	58.73 ± 5.39^{a}	64.42 ± 11.43^{a}	65.83 ± 6.55^{a}	73.30 ± 16.44^{a}	63.98 ± 11.28^{a}	65.25 ± 10.22
**Keratinase [KU cm^{-3}]**						
1.0% (III)	54.77 ± 1.68^{b}	79.57 ± 1.75^{a}	81.03 ± 1.54^{a}	80.73 ± 2.78^{a}	93.70 ± 1.69^{c}	77.96 ± 1.89
1.0% (XII)	40.23 ± 2.05^{a}	38.53 ± 1.55^{a}	48.40 ± 2.27^{b}	44.30 ± 2.60^{ab}	56.97 ± 1.62^{c}	45.69 ± 2.02
1.0% (XIV)	39.67 ± 2.48^{a}	42.87 ± 1.02^{ab}	49.13 ± 2.52^{bc}	50.27 ± 1.62^{c}	56.80 ± 1.39^{d}	47.75 ± 1.81
1.5% (III)	21.13 ± 2.50^{b}	35.73 ± 5.26^{ab}	42.67 ± 7.02^{a}	48.27 ± 5.77^{a}	73.87 ± 5.17^{c}	44.33 ± 5.14
1.5% (XII)	34.03 ± 3.98^{b}	46.70 ± 2.98^{ab}	55.63 ± 9.16^{a}	58.37 ± 3.92^{a}	87.43 ± 3.14^{c}	56.43 ± 4.64
1.5% (XIV)	35.27 ± 0.97^{a}	39.40 ± 1.21^{a}	55.37 ± 3.23^{b}	66.60 ± 2.42^{b}	80.33 ± 6.60^{c}	55.39 ± 2.89
2.0% (III)	34.97 ± 2.05^{b}	60.73 ± 5.74^{a}	63.30 ± 2.55^{a}	85.47 ± 2.68^{c}	111.37 ± 3.66^{d}	71.17 ± 3.34
2.0% (XII)	31.73 ± 1.27^{b}	60.00 ± 3.32^{a}	54.40 ± 4.62^{a}	84.07 ± 3.49^{c}	116.20 ± 4.47^{d}	69.28 ± 3.43
2.0% (XIV)	44.10 ± 3.43^{b}	69.50 ± 5.13^{a}	79.67 ± 2.86^{a}	107.10 ± 2.05^{c}	127.30 ± 8.15^{d}	85.53 ± 4.33
**Disulfide reductase [U cm^{-3}]**						
1.0% (III)	0.010 ± 0.001^{a}	0.013 ± 0.005^{a}	0.013 ± 0.005^{a}	0.019 ± 0.008^{ab}	0.032 ± 0.005^{b}	0.017 ± 0.004
1.0% (XII)	0.194 ± 0.008^{ab}	0.188 ± 0.012^{a}	0.223 ± 0.008^{bc}	0.207 ± 0.012^{ab}	0.256 ± 0.012^{c}	0.214 ± 0.010
1.0% (XIV)	0.013 ± 0.005^{a}	0.036 ± 0.009^{b}	0.019 ± 0.008^{ab}	0.026 ± 0.005^{ab}	0.084 ± 0.005^{c}	0.036 ± 0.006
1.5% (III)	0.013 ± 0.005^{a}	0.019 ± 0.001^{a}	0.026 ± 0.009^{a}	0.023 ± 0.005^{a}	0.029 ± 0.008^{a}	0.022 ± 0.005
1.5% (XII)	0.074 ± 0.005^{b}	0.065 ± 0.005^{b}	0.036 ± 0.005^{a}	0.026 ± 0.009^{a}	0.029 ± 0.001^{a}	0.046 ± 0.005
1.5% (XIV)	0.104 ± 0.012^{b}	0.039 ± 0.008^{a}	0.133 ± 0.009^{c}	0.049 ± 0.008^{a}	0.055 ± 0.005^{a}	0.076 ± 0.008
2.0% (III)	0.023 ± 0.005^{bc}	0.013 ± 0.005^{b}	0.039 ± 0.008^{ac}	0.042 ± 0.005^{a}	0.052 ± 0.005^{a}	0.034 ± 0.005
2.0% (XII)	0.032 ± 0.005^{bc}	0.019 ± 0.008^{b}	0.065 ± 0.005^{a}	0.065 ± 0.009^{a}	0.055 ± 0.009^{ac}	0.047 ± 0.007
2.0% (XIV)	0.061 ± 0.005^{a}	0.016 ± 0.005^{b}	0.107 ± 0.008^{c}	0.058 ± 0.008^{a}	0.061 ± 0.005^{a}	0.061 ± 0.006
**Proteins and peptides [µg of proteins cm^{-3}]**						
1.0% (III)	297.42 ± 5.44^{b}	473.18 ± 7.52^{a}	473.37 ± 6.33^{a}	509.37 ± 7.35^{c}	545.95 ± 19.68^{d}	459.86 ± 9.26
1.0% (XII)	341.12 ± 20.62^{b}	421.78 ± 14.01^{a}	387.32 ± 9.27^{ab}	394.06 ± 14.68^{a}	430.25 ± 15.16^{a}	394.91 ± 14.75
1.0% (XIV)	308.40 ± 7.41^{d}	394.06 ± 1.91^{bc}	365.19 ± 2.37^{a}	378.85 ± 6.48^{ab}	413.12 ± 10.90^{c}	371.92 ± 5.81
1.5% (III)	292.03 ± 24.33^{c}	457.40 ± 26.14^{a}	472.03 ± 17.48^{a}	539.02 ± 12.87^{b}	573.67 ± 11.52^{b}	466.83 ± 18.47
1.5% (XII)	350.55 ± 5.44^{a}	470.10 ± 1.25^{b}	539.79 ± 1.66^{c}	605.82 ± 4.79^{d}	663.57 ± 18.93^{e}	525.97 ± 6.41
1.5% (XIV)	399.45 ± 44.04^{a}	504.56 ± 46.01^{a}	613.13 ± 20.28^{c}	719.78 ± 17.11^{b}	783.12 ± 21.75^{b}	604.01 ± 29.84
2.0% (III)	431.79 ± 12.84^{d}	602.35 ± 3.56^{a}	687.63 ± 31.93^{ab}	791.01 ± 48.45^{bc}	902.47 ± 53.69^{c}	683.05 ± 30.09
2.0% (XII)	378.47 ± 21.26^{b}	585.41 ± 35.92^{c}	673.77 ± 22.88^{d}	773.30 ± 1.70^{a}	836.44 ± 26.70^{a}	649.48 ± 21.69
2.0% (XIV)	469.91 ± 20.01^{b}	686.29 ± 34.06^{c}	773.49 ± 31.41^{d}	853.38 ± 2.88^{a}	921.72 ± 9.29^{a}	740.96 ± 19.53
**Amino groups [µg N-NH$_2$ cm^{-3}]**						
1.0% (III)	5.78 ± 0.43^{a}	10.62 ± 0.23^{b}	10.34 ± 0.30^{b}	6.16 ± 0.33^{a}	6.61 ± 0.19^{a}	7.90 ± 0.30
1.0% (XII)	7.02 ± 0.16^{b}	10.89 ± 0.15^{c}	11.98 ± 0.31^{a}	12.57 ± 0.34^{a}	14.46 ± 0.33^{d}	11.38 ± 0.26
1.0% (XIV)	5.37 ± 0.31^{b}	8.32 ± 0.24^{a}	9.15 ± 0.28^{a}	7.32 ± 0.17^{c}	8.42 ± 0.32^{a}	7.72 ± 0.26
1.5% (III)	3.80 ± 0.16^{b}	13.79 ± 0.72^{c}	31.97 ± 1.02^{a}	32.41 ± 1.83^{a}	33.71 ± 1.86^{a}	23.14 ± 1.12
1.5% (XII)	2.76 ± 0.18^{b}	9.03 ± 0.39^{c}	24.48 ± 0.52^{a}	25.29 ± 1.50^{a}	25.96 ± 0.75^{a}	17.50 ± 0.67
1.5% (XIV)	2.80 ± 0.12^{b}	8.80 ± 0.89^{c}	25.17 ± 0.47^{a}	26.66 ± 0.92^{a}	27.56 ± 1.45^{a}	18.20 ± 0.77
2.0% (III)	2.48 ± 0.10^{b}	12.00 ± 0.57^{c}	31.17 ± 2.14^{a}	27.07 ± 1.73^{a}	27.84 ± 1.14^{a}	20.11 ± 1.14
2.0% (XII)	3.60 ± 0.15^{b}	8.74 ± 0.96^{c}	35.18 ± 0.82^{d}	21.55 ± 1.09^{a}	22.58 ± 1.71^{a}	18.33 ± 0.95
2.0% (XIV)	2.93 ± 0.11^{b}	9.44 ± 0.28^{c}	29.33 ± 0.03^{d}	25.94 ± 0.95^{a}	27.06 ± 0.89^{a}	18.94 ± 0.45

**Thiol groups [μg -SH cm^{-3}]**						
1.0% (III)	2.35 ± 0.15^{a}	2.99 ± 0.15^{ab}	6.72 ± 0.47^{d}	4.69 ± 0.15^{c}	3.95 ± 0.53^{bc}	4.14 ± 0.29
1.0% (XII)	5.28 ± 0.13^{ab}	7.14 ± 0.87^{c}	3.73 ± 0.40^{a}	4.37 ± 0.33^{a}	6.13 ± 0.42^{bc}	5.33 ± 0.43
1.0% (XIV)	4.80 ± 0.23^{a}	5.33 ± 0.33^{a}	4.53 ± 0.87^{a}	4.69 ± 0.53^{a}	4.74 ± 0.96^{a}	4.82 ± 0.58
1.5% (III)	2.24 ± 0.35^{ab}	3.47 ± 0.46^{b}	2.67 ± 0.33^{ab}	1.71 ± 0.27^{a}	7.09 ± 0.79^{c}	3.43 ± 0.44
1.5% (XII)	6.18 ± 0.42^{c}	3.52 ± 0.47^{b}	9.49 ± 0.49^{d}	0.69 ± 0.20^{a}	19.51 ± 1.45^{e}	7.88 ± 0.61
1.5% (XIV)	8.48 ± 0.86^{a}	5.22 ± 0.49^{a}	8.69 ± 1.63^{a}	5.33 ± 0.33^{a}	23.99 ± 2.26^{b}	10.34 ± 1.11
2.0% (III)	7.84 ± 0.13^{a}	8.26 ± 1.11^{ab}	11.04 ± 0.79^{b}	9.92 ± 0.60^{ab}	38.33 ± 1.44^{c}	15.08 ± 0.81
2.0% (XII)	6.02 ± 0.80^{a}	9.44 ± 1.25^{a}	14.82 ± 2.12^{b}	22.44 ± 1.17^{c}	34.76 ± 2.08^{d}	17.50 ± 1.48
2.0% (XIV)	11.04 ± 1.77^{a}	9.49 ± 1.49^{a}	34.17 ± 0.72^{c}	9.97 ± 0.42^{a}	25.96 ± 2.17^{b}	18.13 ± 1.31
**Sulfate ions [mg SO_4^{2-} cm^{-3}]**						
1.0% (III)	0.32 ± 0.03^{a}	0.95 ± 0.02^{d}	1.15 ± 0.02^{e}	0.55 ± 0.03^{b}	0.66 ± 0.03^{c}	0.73 ± 0.02
1.0% (XII)	0.59 ± 0.04^{a}	0.96 ± 0.04^{c}	0.57 ± 0.03^{a}	0.17 ± 0.01^{b}	0.53 ± 0.02^{a}	0.56 ± 0.03
1.0% (XIV)	0.34 ± 0.03^{a}	1.00 ± 0.06^{b}	0.91 ± 0.01^{b}	0.31 ± 0.10^{a}	0.60 ± 0.02^{c}	0.63 ± 0.04
1.5% (III)	0.94 ± 0.04^{b}	0.71 ± 0.03^{a}	1.18 ± 0.02^{c}	0.74 ± 0.03^{a}	0.93 ± 0.03^{b}	0.90 ± 0.03
1.5% (XII)	0.86 ± 0.03^{a}	0.72 ± 0.03^{c}	1.31 ± 0.03^{d}	0.90 ± 0.03^{ab}	1.02 ± 0.06^{b}	0.96 ± 0.04
1.5% (XIV)	0.77 ± 0.01^{a}	0.51 ± 0.02^{b}	1.03 ± 0.03^{c}	0.71 ± 0.04^{a}	0.76 ± 0.04^{a}	0.76 ± 0.03
2.0% (III)	1.10 ± 0.03^{a}	1.06 ± 0.17^{a}	1.75 ± 0.08^{b}	1.12 ± 0.05^{a}	1.27 ± 0.02^{a}	1.26 ± 0.07
2.0% (XII)	1.07 ± 0.02^{a}	1.15 ± 0.03^{ab}	1.94 ± 0.04^{c}	1.10 ± 0.03^{a}	1.26 ± 0.04^{b}	1.30 ± 0.03
2.0% (XIV)	0.95 ± 0.03^{a}	0.78 ± 0.02^{b}	1.57 ± 0.03^{d}	0.98 ± 0.04^{a}	1.12 ± 0.04^{c}	1.08 ± 0.03
**Ammonium ions [μg NH_4^+ cm^{-3}]**						
1.0% (III)	365.53 ± 7.71^{c}	637.65 ± 9.38^{a}	620.27 ± 23.04^{a}	558.61 ± 7.19^{b}	598.95 ± 12.50^{ab}	556.20 ± 11.96
1.0% (XII)	525.24 ± 7.75^{b}	687.03 ± 8.92^{a}	677.99 ± 5.42^{a}	555.37 ± 9.20^{c}	593.61 ± 10.50^{d}	607.85 ± 8.36
1.0% (XIV)	460.33 ± 14.23^{b}	646.23 ± 2.29^{a}	668.71 ± 11.49^{a}	531.49 ± 8.98^{c}	578.08 ± 6.02^{d}	576.97 ± 8.60
1.5% (III)	381.53 ± 15.34^{c}	732.92 ± 28.62^{b}	738.95 ± 29.06^{b}	535.90 ± 3.47^{a}	568.58 ± 32.35^{a}	591.57 ± 21.77
1.5% (XII)	434.37 ± 57.12^{a}	501.59 ± 13.84^{a}	562.09 ± 22.43^{a}	874.31 ± 45.89^{b}	531.49 ± 47.75^{a}	580.77 ± 37.40
1.5% (XIV)	306.19 ± 23.28^{c}	429.51 ± 33.58^{a}	528.02 ± 20.32^{b}	539.38 ± 6.12^{b}	442.95 ± 25.09^{a}	449.21 ± 21.68
2.0% (III)	457.79 ± 29.37^{b}	638.35 ± 46.84^{a}	721.79 ± 65.36^{a}	621.20 ± 49.91^{a}	622.82 ± 44.88^{a}	612.39 ± 47.27
2.0% (XII)	290.66 ± 36.08^{c}	735.01 ± 69.05^{ab}	813.35 ± 23.34^{b}	679.84 ± 16.47^{a}	643.22 ± 6.32^{a}	632.42 ± 30.25
2.0% (XIV)	186.36 ± 19.90^{c}	583.65 ± 29.06^{ab}	669.18 ± 18.15^{b}	595.70 ± 42.34^{ab}	530.10 ± 69.55^{a}	513.00 ± 35.80

Explanations: as in Table S1.

Table S3. Dynamics of changes in enzymatic activity and the secretion of organic and mineral products of chicken feathers keratinolysis by *Trichophyton ajelloi* strains at different initial pH (4.5, 6.5, 8.5) of the culture medium.

Days	7	14	21	28	35	Mean
**Protease [μg tyrosine cm^{-3}]**						
4.5 (III)	48.15 ± 3.90^{a}	58.87 ± 3.26^{b}	40.24 ± 0.55^{a}	59.02 ± 3.99^{b}	70.56 ± 1.27^{c}	55.37 ± 2.59
4.5 (XII)	44.82 ± 1.09^{ac}	47.56 ± 2.08^{a}	37.57 ± 1.64^{b}	40.01 ± 1.27^{bc}	49.41 ± 2.16^{a}	43.88 ± 1.65
4.5 (XIV)	49.04 ± 2.54^{c}	38.09 ± 3.02^{ab}	32.84 ± 0.79^{a}	33.14 ± 3.26^{a}	42.16 ± 1.57^{bc}	39.05 ± 2.24
6.5 (III)	95.34 ± 5.96^{b}	55.18 ± 7.28^{a}	39.27 ± 3.72^{a}	37.06 ± 5.97^{a}	43.64 ± 6.28^{a}	54.10 ± 5.84
6.5 (XII)	48.59 ± 5.33^{a}	37.43 ± 7.00^{a}	34.10 ± 9.48^{a}	42.31 ± 9.31^{a}	43.12 ± 8.59^{a}	41.11 ± 7.94
6.5 (XIV)	24.33 ± 2.77^{ab}	21.30 ± 2.27^{ab}	19.16 ± 1.05^{a}	23.30 ± 1.31^{ab}	26.40 ± 1.84^{b}	22.90 ± 1.85
8.5 (III)	51.70 ± 9.83^{a}	58.87 ± 9.80^{a}	34.61 ± 6.73^{a}	35.95 ± 4.87^{a}	40.38 ± 3.46^{a}	44.30 ± 6.94
8.5 (XII)	26.40 ± 2.74^{a}	29.29 ± 3.92^{a}	30.32 ± 5.00^{a}	34.10 ± 1.11^{a}	36.02 ± 3.12^{a}	31.23 ± 3.18
8.5 (XIV)	18.49 ± 3.72^{a}	18.05 ± 2.61^{a}	23.96 ± 4.26^{a}	27.00 ± 2.28^{a}	25.59 ± 3.40^{a}	22.62 ± 3.26
**Keratinase [KU cm^{-3}]**						
4.5 (III)	54.77 ± 1.68^{b}	79.57 ± 1.75^{a}	81.03 ± 1.54^{a}	80.73 ± 2.78^{a}	93.70 ± 1.69^{c}	77.96 ± 1.89
4.5 (XII)	40.23 ± 2.05^{a}	38.53 ± 1.55^{a}	48.40 ± 2.27^{b}	44.30 ± 2.60^{ab}	56.97 ± 1.62^{c}	45.69 ± 2.02
4.5 (XIV)	39.67 ± 2.48^{a}	42.87 ± 1.02^{ab}	49.13 ± 2.52^{bc}	50.27 ± 1.62^{c}	56.80 ± 1.39^{d}	47.75 ± 1.81
6.5 (III)	21.87 ± 3.97^{a}	29.40 ± 6.13^{ab}	44.93 ± 6.84^{bc}	106.70 ± 4.32^{d}	60.77 ± 4.15^{c}	52.73 ± 5.08
6.5 (XII)	16.97 ± 0.87^{b}	33.23 ± 1.32^{a}	43.13 ± 3.01^{a}	97.83 ± 5.27^{d}	60.40 ± 3.66^{c}	50.31 ± 2.83
6.5 (XIV)	15.80 ± 3.64^{a}	14.33 ± 2.39^{a}	34.20 ± 0.80^{b}	90.00 ± 3.63^{d}	49.10 ± 2.05^{c}	40.69 ± 2.50
8.5 (III)	18.17 ± 1.20^{a}	18.80 ± 6.61^{a}	39.13 ± 5.61^{b}	98.03 ± 2.99^{d}	62.38 ± 3.33^{c}	47.30 ± 3.95
8.5 (XII)	18.97 ± 1.81^{a}	16.70 ± 2.33^{a}	42.27 ± 5.38^{b}	115.33 ± 2.49^{d}	64.07 ± 3.07^{c}	51.47 ± 3.01
8.5 (XIV)	19.70 ± 7.02^{a}	16.47 ± 4.25^{a}	36.93 ± 4.69^{b}	110.23 ± 4.66^{d}	63.27 ± 2.67^{c}	49.32 ± 4.66
**Disulfide reductase [U cm^{-3}]**						
4.5 (III)	0.010 ± 0.001^{a}	0.013 ± 0.005^{a}	0.013 ± 0.005^{a}	0.019 ± 0.008^{ab}	0.032 ± 0.005^{b}	0.017 ± 0.004
4.5 (XII)	0.194 ± 0.008^{ab}	0.188 ± 0.012^{a}	0.223 ± 0.008^{bc}	0.207 ± 0.012^{ab}	0.256 ± 0.012^{c}	0.214 ± 0.010
4.5 (XIV)	0.013 ± 0.005^{a}	0.036 ± 0.009^{b}	0.019 ± 0.008^{ab}	0.026 ± 0.005^{ab}	0.084 ± 0.005^{c}	0.036 ± 0.006
6.5 (III)	0.019 ± 0.008^{a}	0.036 ± 0.009^{ab}	0.029 ± 0.008^{ab}	0.049 ± 0.008^{b}	0.084 ± 0.009^{c}	0.043 ± 0.008
6.5 (XII)	0.039 ±0.008^{a}	0.045 ± 0.005^{a}	0.026 ± 0.005^{a}	0.078 ± 0.008b	0.094 ± 0.012^{b}	0.056 ± 0.007
6.5 (XIV)	0.029 ± 0.008^{ab}	0.042 ± 0.012^{ab}	0.036 ± 0.009^{ab}	0.023 ± 0.005^{a}	0.055 ± 0.012^{b}	0.037 ± 0.009
8.5 (III)	0.016 ± 0.005^{b}	0.055 ± 0.009^{a}	0.045 ± 0.005^{a}	0.058 ± 0.008^{a}	0.149 ± 0.012^{c}	0.065 ± 0.008
8.5 (XII)	0.013 ± 0.005^{a}	0.029 ± 0.008^{ab}	0.042 ± 0.005^{b}	0.016 ± 0.005^{a}	0.117 ± 0.008^{c}	0.043 ± 0.006
8.5 (XIV)	0.026 ± 0.017^{a}	0.029 ± 0.008^{a}	0.039 ± 0.014^{a}	0.039 ± 0.008^{a}	0.091 ± 0.012^{b}	0.045 ± 0.012
**Proteins and peptides [μg of proteins cm^{-3}]**						
4.5 (III)	297.42 ± 5.44^{b}	473.18 ± 7.52^{a}	473.37 ± 6.33^{a}	509.37 ± 7.35^{c}	545.95 ± 19.68^{d}	459.86 ± 9.26
4.5 (XII)	341.12 ± 20.62^{b}	421.78 ± 14.01^{a}	387.32 ± 9.27^{ab}	394.06 ± 14.68^{a}	430.25 ± 15.16^{a}	394.91 ± 14.75
4.5 (XIV)	308.40 ± 7.41^{d}	394.06 ± 1.91^{bc}	365.19 ± 2.37^{a}	378.85 ± 6.48^{ab}	413.12 ± 10.90^{c}	371.92 ± 5.81
6.5 (III)	253.15 ± 14.66^{c}	331.88 ± 15.36^{a}	372.89 ± 9.63^{a}	457.78 ± 26.16^{b}	499.55 ± 13.07^{b}	383.05 ± 15.78
6.5 (XII)	222.54 ± 31.28^{a}	319.95 ± 16.78^{ab}	385.40 ± 24.89^{bc}	478.38 ± 25.49^{cd}	500.90 ± 48.65^{d}	381.43 ± 29.42
6.5 (XIV)	176.72 ± 15.18^{a}	258.34 ± 16.51^{b}	313.98 ± 12.95^{c}	401.38 ± 10.99^{d}	445.85 ± 9.68^{e}	319.25 ± 13.06
8.5 (III)	182.69 ± 24.00^{c}	275.67 ± 23.98^{a}	332.65 ± 11.62^{ab}	385.21 ± 21.24^{b}	465.10 ± 1.96^{d}	328.26 ± 16.56
8.5 (XII)	192.12 ± 26.88^{c}	316.48 ± 6.95^{a}	354.02 ± 13.88^{a}	436.80 ± 26.07^{b}	490.31 ± 16.40^{b}	357.95 ± 18.04
8.5 (XIV)	166.90 ± 28.06^{a}	262.19 ± 14.73^{ab}	317.64 ± 42.48^{bc}	401.95 ± 41.16^{cd}	454.51 ± 45.75^{d}	320.64 ± 34.44
**Amino groups [μg N-NH$_2$ cm^{-3}]**						
4.5 (III)	5.78 ± 0.43^{a}	10.62 ± 0.23^{b}	10.34 ± 0.30^{b}	6.16 ± 0.33^{a}	6.61 ± 0.19^{a}	7.90 ± 0.30
4.5 (XII)	7.02 ± 0.16^{b}	10.89 ± 0.15^{c}	11.98 ± 0.31^{a}	12.57 ± 0.34^{a}	14.46 ± 0.33^{d}	11.38 ± 0.26
4.5 (XIV)	5.37 ± 0.31^{b}	8.32 ± 0.24^{a}	9.15 ± 0.28^{a}	7.32 ± 0.17^{c}	8.42 ± 0.32^{a}	7.72 ± 0.26
6.5 (III)	24.07 ± 0.68^{a}	32.62 ± 1.06^{b}	50.03 ± 0.70^{d}	36.84 ± 1.80^{c}	23.61 ± 0.39^{a}	33.43 ± 0.92
6.5 (XII)	19.88 ± 0.47^{b}	30.68 ± 1.07^{c}	58.49 ± 0.83^{d}	26.27 ± 0.33^{a}	25.52 ± 1.00^{a}	32.17 ± 0.74
6.5 (XIV)	20.66 ± 0.14^{a}	30.69 ± 1.10^{b}	55.85 ± 1.54^{c}	27.76 ± 1.00^{b}	22.50 ± 0.49^{a}	31.49 ± 0.85
8.5 (III)	16.18 ± 0.71^{a}	25.00 ± 0.24^{b}	45.72 ± 2.00^{c}	19.25 ± 1.25^{a}	16.60 ± 1.07^{a}	24.55 ± 1.06
8.5 (XII)	15.87 ± 0.90^{a}	25.27 ± 0.92^{b}	44.97 ± 1.36^{c}	26.01 ± 0.98^{b}	17.77 ± 0.96^{a}	25.98 ± 1.03
8.5 (XIV)	18.61 ± 0.43^{a}	23.49 ± 0.28^{b}	36.28 ± 0.59^{d}	28.56 ± 0.71^{c}	19.34 ± 0.44^{a}	25.26 ±0.49

**Thiol groups [μg -SH cm^{-3}]**						
4.5 (III)	2.35 ± 0.15^{a}	2.99 ± 0.15^{ab}	6.72 ± 0.47^{d}	4.69 ± 0.15^{c}	3.95 ± 0.53^{bc}	4.14 ± 0.29
4.5 (XII)	5.28 ± 0.13^{ab}	7.14 ± 0.87^{c}	3.73 ± 0.40^{a}	4.37 ± 0.33^{a}	6.13 ± 0.42^{bc}	5.33 ± 0.43
4.5 (XIV)	4.80 ± 0.23^{a}	5.33 ± 0.33^{a}	4.53 ± 0.87^{a}	4.69 ± 0.53^{a}	4.74 ± 0.96^{a}	4.82 ± 0.58
6.5 (III)	3.84 ± 0.26^{a}	0.85 ± 0.20^{b}	0.80 ± 0.13^{b}	4.69 ± 1.06^{a}	4.90 ± 1.29^{a}	3.02 ± 0.59
6.5 (XII)	1.97 ± 0.38^{a}	1.92 ± 0.35^{a}	3.04 ± 0.23^{a}	2.51 ± 0.40^{a}	6.77 ± 0.98^{b}	3.24 ± 0.47
6.5 (XIV)	0.75 ± 0.20^{a}	1.71 ± 0.53^{a}	3.89 ± 0.59^{b}	1.49 ± 0.42^{a}	2.24 ± 0.47^{a}	2.02 ± 0.44
8.5 (III)	2.72 ± 0.78^{a}	0.91 ± 0.20^{a}	0.69 ± 0.20^{a}	2.77 ± 0.53^{a}	5.81 ± 1.18^{b}	2.58 ± 0.58
8.5 (XII)	2.61 ± 0.85^{a}	1.33 ± 0.40^{a}	1.23 ± 0.33^{a}	1.71 ± 0.49^{a}	4.80 ± 0.35^{b}	2.34 ± 0.48
8.5 (XIV)	1.55 ± 0.33^{a}	0.80 ± 0.13^{ab}	1.65 ± 0.08^{a}	0.27 ± 0.15^{b}	5.22 ± 0.59^{c}	1.90± 0.25
**Sulfate ions [mg SO_4^{2-} cm^{-3}]**						
4.5 (III)	0.32 ± 0.03^{a}	0.95 ± 0.02^{d}	1.15 ± 0.02^{e}	0.55 ± 0.03^{b}	0.66 ± 0.03^{c}	0.73 ± 0.02
4.5 (XII)	0.59 ± 0.04^{a}	0.96 ± 0.04^{c}	0.57 ± 0.03^{a}	0.17 ± 0.01^{b}	0.53 ± 0.02^{a}	0.56 ± 0.03
4.5 (XIV)	0.34 ± 0.03^{a}	1.00 ± 0.06^{b}	0.91 ± 0.01^{b}	0.31 ± 0.10^{a}	0.60 ± 0.02^{c}	0.63 ± 0.04
6.5 (III)	0.61 ± 0.03^{b}	0.95 ± 0.02^{a}	0.61 ± 0.03^{b}	0.88 ± 0.04^{a}	0.95 ± 0.03^{a}	0.80 ± 0.03
6.5 (XII)	0.65 ± 0.03^{a}	1.08 ± 0.03^{c}	0.67 ± 0.03^{a}	0.90 ± 0.06^{b}	0.97 ± 0.03^{bc}	0.85 ± 0.04
6.5 (XIV)	0.65 ± 0.03^{a}	1.00 ± 0.04^{b}	0.58 ± 0.03^{a}	0.67 ± 0.02^{a}	1.02 ± 0.04^{b}	0.78 ± 0.03
8.5 (III)	0.67 ± 0.02^{a}	0.98 ± 0.02^{b}	0.55 ± 0.04^{c}	0.73 ± 0.04^{a}	0.92 ± 0.03^{b}	0.77 ± 0.03
8.5 (XII)	0.67 ± 0.02^{a}	0.92 ± 0.04^{b}	0.54 ± 0.03^{c}	0.73 ± 0.02^{a}	0.88 ± 0.04^{b}	0.75 ± 0.03
8.5 (XIV)	0.73 ± 0.01^{ab}	0.82 ± 0.02^{a}	0.67 ± 0.04^{b}	0.80 ± 0.03^{a}	0.94 ± 0.04^{c}	0.79 ± 0.03
**Ammonium ions [μg NH^{4+} cm^{-3}]**						
4.5 (III)	365.53 ± 7.71^{c}	637.65 ± 9.38^{a}	620.27 ± 23.04^{a}	558.61 ± 7.19^{b}	598.95 ± 12.50^{ab}	556.20 ± 11.96
4.5 (XII)	525.24 ± 7.75^{b}	687.03 ± 8.92^{a}	677.99 ± 5.42^{a}	555.37 ± 9.20^{c}	593.61 ± 10.50^{d}	607.85 ± 8.36
4.5 (XIV)	460.33 ± 14.23^{b}	646.23 ± 2.29^{a}	668.71 ± 11.49^{a}	531.49 ± 8.98^{c}	578.08 ± 6.02^{d}	576.97 ± 8.60
6.5 (III)	459.41 ± 27.58^{a}	587.36 ± 40.84^{c}	544.24 ± 16.69^{bc}	482.59 ± 9.85^{ab}	431.82 ± 16.78^{a}	501.08 ± 22.35
6.5 (XII)	474.01 ± 13.16^{ab}	574.61 ± 41.73^{c}	528.71 ± 6.25^{bc}	474.01 ± 16.55^{ab}	441.56 ± 15.56^{a}	498.58 ± 18.65
6.5 (XIV)	445.27 ± 12.51^{a}	565.80 ± 19.51^{c}	524.31 ± 28.23^{bc}	464.97 ± 19.48^{ab}	414.90 ± 9.02^{a}	483.05 ± 17.75
8.5 (III)	327.06 ± 39.48^{a}	491.86 ± 16.06^{c}	437.85 ± 19.87^{bc}	364.61 ± 13.63^{ab}	295.07 ± 17.05^{a}	383.29 ± 21.22
8.5 (XII)	363.45 ± 41.48^{a}	493.25 ± 11.97^{b}	439.94 ± 21.05^{b}	348.84 ± 10.00^{a}	296.92 ± 10.79^{a}	388.48 ± 19.06
8.5 (XIV)	338.41 ± 28.50^{a}	475.17 ± 8.80^{b}	427.88 ± 16.55^{b}	349.77 ± 11.40^{a}	294.61 ± 15.67^{a}	377.17 ± 16.18

Explanations: as in Table S1.

Table S4. Dynamics of changes in enzymatic activity and the secretion of organic and mineral products of chicken feathers keratinolysis of by *Trichophyton ajelloi* strains fungi in various culture temperature variants (20, 28 and 37°C).

Days	7	14	21	28	35	Mean
**Protease [µg tyrosine cm^{-3}]**						
20°C (III)	61.69 ± 12.15^{d}	246.82 ± 8.67^{c}	226.48 ± 6.42^{a}	188.46 ± 22.34^{ab}	173.00 ± 12.58^{a}	179.29 ± 12.43
20°C (XII)	51.18 ± 8.25^{c}	240.90 ± 5.80^{b}	211.24 ± 3.54^{b}	163.31 ± 18.21^{a}	134.76 ± 15.21^{a}	160.28 ± 10.20
20°C (XIV)	168.93 ± 0.75^{a}	221.30 ± 4.86^{b}	204.36 ± 9.91^{b}	159.76 ± 5.99^{a}	136.17 ± 8.70^{c}	178.10 ± 6.04
28°C (III)	48.15 ± 3.90^{a}	58.87 ± 3.26^{b}	40.24 ± 0.55^{a}	59.02 ± 3.99^{b}	70.56 ± 1.27^{c}	55.37 ± 2.59
28°C (XII)	44.82 ± 1.09^{ac}	47.56 ± 2.08^{a}	37.57 ± 1.64^{b}	40.01 ± 1.27^{bc}	49.41 ± 2.16^{a}	43.88 ± 1.65
28°C (XIV)	49.04 ± 2.54^{c}	38.09 ± 3.02^{ab}	32.84 ± 0.79^{a}	33.14 ± 3.26^{a}	42.16 ± 1.57^{bc}	39.05 ± 2.24
37°C (III)	24.11 ± 0.42^{a}	26.63 ± 2.06^{ab}	35.72 ± 2.37^{b}	27.88 ± 4.45^{ab}	31.06 ± 3.36^{ab}	29.08 ± 2.53
37°C (XII)	22.63 ± 1.89^{a}	27.07 ± 1.89^{a}	41.05 ± 7.62^{b}	30.77 ± 2.91^{ab}	33.51 ± 3.03^{ab}	31.01 ± 3.47
37°C (XIV)	14.27 ± 0.89^{a}	22.41 ± 2.57^{ab}	25.07 ± 3.64^{ab}	23.45 ± 7.01^{ab}	27.07 ± 2.04^{b}	22.46 ± 3.23
**Keratinase [KU cm^{-3}]**						
20°C (III)	24.47 ± 4.58^{b}	37.00 ± 4.93^{ab}	40.20 ± 3.69^{a}	50.20 ± 4.34^{ac}	63.17 ± 4.83^{c}	43.01 ± 4.47
20°C (XII)	20.77 ± 3.84^{b}	38.07 ± 7.84^{a}	38.47 ± 6.71^{a}	42.07 ± 1.51^{a}	50.70 ± 3.48^{a}	38.01 ± 4.68
20°C (XIV)	24.70 ± 3.16^{a}	39.33 ± 2.37^{bc}	31.57 ± 4.83^{ab}	43.13 ± 2.11^{c}	56.63 ± 2.32^{d}	39.07 ± 2.96
28°C (III)	54.77 ± 1.68^{b}	79.57 ± 1.75^{a}	81.03 ± 1.54^{a}	80.73 ± 2.78^{a}	93.70 ± 1.69^{c}	77.96 ± 1.89
28°C (XII)	40.23 ± 2.05^{a}	38.53 ± 1.55^{a}	48.40 ± 2.27^{b}	44.30 ± 2.60^{ab}	56.97 ± 1.62^{c}	45.69 ± 2.02
28°C (XIV)	39.67 ± 2.48^{a}	42.87 ± 1.02^{ab}	49.13 ± 2.52^{bc}	50.27 ± 1.62^{c}	56.80 ± 1.39^{d}	47.75 ± 1.81
37°C (III)	34.87 ± 3.30^{ab}	29.13 ± 6.30^{a}	28.17 ± 3.92^{a}	45.70 ± 3.47^{bc}	56.67 ± 3.33^{c}	38.91 ± 4.06
37°C (XII)	37.47 ± 2.90^{a}	31.73 ± 1.81^{ab}	24.83 ± 0.80^{b}	39.53 ± 3.25^{a}	66.70 ± 3.29^{c}	40.05 ± 2.41
37°C (XIV)	36.80 ± 1.95^{a}	31.30 ± 4.08^{ab}	16.53 ± 3.59^{b}	35.73 ± 8.70^{a}	44.67 ± 1.92^{a}	33.01 ± 4.05
**Disulfide reductase [U cm^{-3}]**						
20°C (III)	0.010 ± 0.001^{a}	0.019 ± 0.008^{ab}	0.010 ± 0.001^{a}	0.016 ± 0.006^{ab}	0.026 ± 0.005^{b}	0.016 ± 0.003
20°C (XII)	0.013 ± 0.005^{a}	0.013 ± 0.005^{a}	0.019 ± 0.001^{a}	0.019 ± 0.008^{a}	0.013 ± 0.005^{a}	0.016 ± 0.004
20°C (XIV)	0.010 ± 0.001^{a}	0.013 ± 0.005^{a}	0.013 ± 0.005^{a}	0.013 ± 0.005^{a}	0.032 ± 0.005^{b}	0.016 ± 0.004
28°C (III)	0.010 ± 0.001^{a}	0.013 ± 0.005^{a}	0.013 ± 0.005^{a}	0.019 ± 0.008^{ab}	0.032 ± 0.005^{b}	0.017 ± 0.004
28°C (XII)	0.194 ± 0.008^{ab}	0.188 ± 0.012^{a}	0.223 ± 0.008^{bc}	0.207 ± 0.012^{ab}	0.256 ± 0.012^{c}	0.214 ± 0.010
28°C (XIV)	0.013 ± 0.005^{a}	0.036 ± 0.009^{b}	0.019 ± 0.008^{ab}	0.026 ± 0.005^{ab}	0.084 ± 0.005^{c}	0.036 ± 0.006
37°C (III)	0.016 ± 0.005^{a}	0.013 ± 0.005^{a}	0.016 ± 0.005^{a}	0.049 ± 0.008^{b}	0.016 ± 0.005^{a}	0.022 ± 0.005
37°C (XII)	0.045 ± 0.005^{a}	0.013 ± 0.005^{b}	0.055 ± 0.009^{a}	0.045 ± 0.005^{a}	0.074 ± 0.005^{c}	0.047 ± 0.005
37°C (XIV)	0.023 ± 0.005^{a}	0.013 ± 0.005^{a}	0.026 ± 0.005^{ab}	0.042 ± 0.005^{b}	0.016 ± 0.009^{a}	0.024 ± 0.005
**Proteins and peptides [µg of proteins cm^{-3}]**						
20°C (III)	214.84 ± 10.15^{c}	397.91 ± 21.41^{a}	434.49 ± 28.52^{a}	516.69 ± 13.52^{b}	507.83 ± 28.80^{b}	414.35 ± 20.48
20°C (XII)	228.31 ± 2.84^{b}	460.28 ± 20.86^{a}	442.96 ± 35.95^{a}	503.40 ± 29.84^{a}	499.94 ± 26.64^{a}	426.9 ± 23.23
20°C (XIV)	259.88 ± 29.34^{c}	393.87 ± 24.32^{b}	435.26 ± 17.48^{ab}	491.28 ± 17.03^{a}	488.58 ± 1.25^{a}	413.77 ± 17.88
28°C (III)	297.42 ± 5.44^{b}	473.18 ± 7.52^{a}	473.37 ± 6.33^{a}	509.37 ± 7.35^{c}	545.95 ± 19.68^{d}	459.86 ± 9.26
28°C (XII)	341.12 ± 20.62^{b}	421.78 ± 14.01^{a}	387.32 ± 9.27^{ab}	394.06 ± 14.68^{a}	430.25 ± 15.16^{a}	394.91 ± 14.75
28°C (XIV)	308.40 ± 7.41^{d}	394.06 ± 1.91^{bc}	365.19 ± 2.37^{a}	378.85 ± 6.48^{ab}	413.12 ± 10.90^{c}	371.92 ± 5.81
37°C (III)	333.61 ± 17.53^{b}	413.89 ± 12.21^{ab}	455.47 ± 19.88^{a}	488.97 ± 50.49^{ac}	575.79 ± 36.55^{c}	453.55 ± 27.33
37°C (XII)	316.87 ± 18.58^{a}	381.55 ± 14.84^{a}	463.56 ± 12.80^{b}	522.08 ± 16.58^{bc}	572.51 ± 31.23^{c}	451.31 ± 18.81
37°C (XIV)	365.96 ± 24.33^{a}	424.28 ± 52.95^{a}	458.17 ± 31.46^{ab}	539.79 ± 29.74^{bc}	596.19 ± 26.27^{c}	476.88 ± 32.95
**Amino groups [µg N-NH_2 cm^{-3}]**						
20°C (III)	2.89 ± 0.12^{b}	12.06 ± 0.34^{c}	32.57 ± 0.11^{a}	30.08 ± 1.23^{a}	31.45 ± 1.19^{a}	21.81 ± 0.59
20°C (XII)	3.27 ± 0.16^{b}	14.01 ± 0.73^{c}	32.57 ± 0.78^{d}	25.68 ± 2.06^{a}	26.21 ± 1.47^{a}	20.35 ± 1.04
20°C (XIV)	3.04 ± 0.17^{b}	13.05 ± 0.53^{c}	32.03 ± 1.37^{a}	31.30 ± 0.92^{a}	32.51 ± 1.39^{a}	22.39 ± 0.87
28°C (III)	5.78 ± 0.43^{a}	10.62 ± 0.23^{b}	10.34 ± 0.30^{b}	6.16 ± 0.33^{a}	6.61 ± 0.19^{a}	7.90 ± 0.30
28°C (XII)	7.02 ± 0.16^{b}	10.89 ± 0.15^{c}	11.98 ± 0.31^{a}	12.57 ± 0.34^{a}	14.46 ± 0.33^{d}	11.38 ± 0.26
28°C (XIV)	5.37 ± 0.31^{b}	8.32 ± 0.24^{a}	9.15 ± 0.28^{a}	7.32 ± 0.17^{c}	8.42 ± 0.32^{a}	7.72 ± 0.26
37°C (III)	3.34 ± 0.08^{ac}	1.87 ± 0.07^{c}	4.43 ± 0.62^{ab}	5.03 ± 0.25^{ab}	5.73 ± 1.03^{b}	4.08 ± 0.41
37°C (XII)	3.40 ± 0.14^{a}	1.94 ± 0.13^{a}	5.39 ± 0.49^{b}	6.66 ± 0.65^{bc}	7.07 ± 0.70^{c}	4.89 ± 0.42
37°C (XIV)	2.52 ± 0.15^{a}	1.38 ± 0.04^{a}	4.04 ± 0.20^{b}	4.88 ± 0.69^{bc}	5.41 ± 0.26^{c}	3.64 ± 0.27

**Thiol groups [µg -SH cm^{-3}]**						
20°C (III)	1.60 ± 0.39^{a}	3.41 ± 0.42^{b}	1.92 ± 0.39^{a}	3.89 ± 0.20^{b}	0.96 ± 0.13^{a}	2.36 ± 0.31
20°C (XII)	4.58 ± 1.06^{a}	3.36 ± 0.86^{a}	1.97 ± 0.49^{a}	4.85 ± 0.87^{a}	9.28 ± 1.25^{b}	4.81 ± 0.91
20°C (XIV)	1.39 ± 0.20^{ab}	2.24 ± 0.13^{a}	0.91 ± 0.38^{b}	5.76 ± 0.52^{c}	2.45 ± 0.46^{a}	2.55 ± 0.34
28°C (III)	2.35 ± 0.15^{a}	2.99 ± 0.15^{ab}	6.72 ± 0.47^{d}	4.69 ± 0.15^{c}	3.95 ± 0.53^{bc}	4.14 ± 0.29
28°C (XII)	5.28 ± 0.13^{ab}	7.14 ± 0.87^{c}	3.73 ± 0.40^{a}	4.37 ± 0.33^{a}	6.13 ± 0.42^{bc}	5.33 ± 0.43
28°C (XIV)	4.80 ± 0.23^{a}	5.33 ± 0.33^{a}	4.53 ± 0.87^{a}	4.69 ± 0.53^{a}	4.74 ± 0.96^{a}	4.82 ± 0.58
37°C (III)	3.68 ± 0.35^{a}	2.35 ± 0.33^{a}	1.76 ± 0.26^{a}	9.01 ± 0.20^{b}	35.83 ± 1.51^{c}	10.52 ± 0.53
37°C (XII)	6.13 ± 0.59^{a}	5.81 ± 0.27^{a}	3.57 ± 0.27^{b}	9.33 ± 0.79^{c}	37.59 ± 0.91^{d}	12.49 ± 0.57
37°C (XIV)	3.52 ± 1.26^{a}	4.85 ± 1.18^{a}	20.05 ± 0.74^{b}	16.10 ± 1.10^{b}	31.67 ± 1.71^{c}	15.24 ± 1.20
**Sulfate ions [mg SO_4^{2-} cm^{-3}]**						
20°C (III)	0.73 ± 0.01^{c}	0.64 ± 0.01^{a}	1.18 ± 0.02^{d}	0.66 ± 0.02^{ab}	0.70 ± 0.02^{bc}	0.78 ± 0.01
20°C (XII)	0.49 ± 0.02^{a}	0.70 ± 0.03^{b}	1.23 ± 0.03^{e}	0.95 ± 0.03^{d}	0.83 ± 0.02^{c}	0.84 ± 0.03
20°C (XIV)	0.90 ± 0.05^{c}	0.60 ± 0.02^{b}	1.16 ± 0.02^{d}	0.79 ± 0.03^{a}	0.75 ± 0.02^{a}	0.84 ± 0.03
28°C (III)	0.32 ± 0.03^{a}	0.95 ± 0.02^{d}	1.15 ± 0.02^{e}	0.55 ± 0.03^{b}	0.66 ± 0.03^{c}	0.73 ± 0.02
28°C (XII)	0.59 ± 0.04^{a}	0.96 ± 0.04^{c}	0.57 ± 0.03^{a}	0.17 ± 0.01^{b}	0.53 ± 0.02^{a}	0.56 ± 0.03
28°C (XIV)	0.34 ± 0.03^{a}	1.00 ± 0.06^{b}	0.91 ± 0.01^{b}	0.31 ± 0.10^{a}	0.60 ± 0.02^{c}	0.63 ± 0.04
37°C (III)	0.34 ± 0.02^{b}	0.11 ± 0.03^{a}	0.19 ± 0.02^{a}	0.16 ± 0.03^{a}	0.17 ± 0.02^{a}	0.19 ± 0.02
37°C (XII)	0.34 ± 0.01^{b}	0.08 ± 0.02^{a}	0.14 ± 0.02^{a}	0.17 ± 0.03^{a}	0.16 ± 0.04^{a}	0.18 ± 0.02
37°C (XIV)	0.26 ± 0.02^{c}	0.01 ± 0.01^{a}	0.05 ± 0.01^{ab}	0.05 ± 0.02^{ab}	0.07 ± 0.01^{b}	0.09 ± 0.01
**Ammonium ions [µg NH^{4+} cm^{-3}]**						
20°C (III)	237.82 ± 16.54^{b}	636.50 ± 35.28^{a}	718.09 ± 45.27^{a}	637.65 ± 41.74a	641.83 ± 18.69^{a}	574.38 ± 31.51
20°C (XII)	129.57 ± 44.24^{b}	525.24 ± 32.09^{a}	615.17 ± 42.31^{a}	523.61 ± 3.97^{a}	531.26 ± 20.45^{a}	464.97 ± 28.61
20°C (XIV)	296.00 ± 2.29^{c}	675.67 ± 37.62^{ab}	729.91 ± 8.54^{b}	655.97 ± 32.57^{ab}	635.10 ± 13.16^{a}	598.53 ± 18.84
28°C (III)	365.53 ± 7.71^{c}	637.65 ± 9.38^{a}	620.27 ± 23.04^{a}	558.61 ± 7.19^{b}	598.95 ± 12.50^{ab}	556.20 ± 11.96
28°C (XII)	525.24 ± 7.75^{b}	687.03 ± 8.92^{a}	677.99 ± 5.42^{a}	555.37 ± 9.20^{c}	593.61 ± 10.50^{d}	607.85 ± 8.36
28°C (XIV)	460.33 ± 14.23^{b}	646.23 ± 2.29^{a}	668.71 ± 11.49^{a}	531.49 ± 8.98^{c}	578.08 ± 6.02^{d}	576.97 ± 8.60
37°C (III)	66.52 ± 3.69^{a}	111.95 ± 9.01^{a}	110.80 ± 20.16^{a}	98.05 ± 9.95^{a}	188.21 ± 20.96^{b}	115.11 ± 12.75
37°C (XII)	66.29 ± 7.19^{a}	109.40 ± 4.76^{bc}	138.38 ± 10.36^{c}	89.93 ± 10.50^{ab}	180.80 ± 10.09^{d}	116.96 ± 8.58
37°C (XIV)	12.28 ± 1.73^{b}	28.28 ± 3.78^{ab}	30.60 ± 4.54^{ab}	41.26 ± 12.62^{a}	46.13 ± 3.28^{a}	31.71 ± 5.19

Explanations: as in Table S1.

Table S5. Dynamics of changes in enzymatic activity and the secretion of organic and mineral products of chicken feathers keratinolysis of by *Trichophyton ajelloi* strains with different culture methods.

Days	7	14	21	28	35	Mean
**Protease [μg tyrosine cm^{-3}]**						
ST (III)	48.15 ± 3.90^{a}	58.87 ± 3.26^{b}	40.24 ± 0.55^{a}	59.02 ± 3.99^{b}	70.56 ± 1.27^{c}	55.37 ± 2.59
ST (XII)	44.82 ± 1.09^{ac}	47.56 ± 2.08^{a}	37.57 ± 1.64^{b}	40.01 ± 1.27^{bc}	49.41 ± 2.16^{a}	43.88 ± 1.65
ST (XIV)	49.04 ± 2.54^{c}	38.09 ± 3.02^{ab}	32.84 ± 0.79^{a}	33.14 ± 3.26^{a}	42.16 ± 1.57^{bc}	39.05 ± 2.24
AG (III)	30.62 ± 1.78^{a}	38.61 ± 7.53^{a}	29.51 ± 1.01^{a}	28.85 ± 2.68^{a}	36.32 ± 9.96^{a}	32.78 ± 4.59
AG (XII)	72.48 ± 9.42^{c}	45.41 ± 2.47^{b}	29.66 ± 1.68^{ab}	39.94 ± 8.04^{ab}	23.00 ± 1.83^{a}	42.10 ± 4.69
AG (XIV)	34.61 ± 2.02^{a}	28.99 ± 0.75^{a}	21.75 ± 1.75^{b}	15.53 ± 0.83^{b}	31.43 ± 3.73^{a}	26.46 ± 1.82
**Keratinase [KU cm^{-3}]**						
ST (III)	54.77 ± 1.68^{b}	79.57 ± 1.75^{a}	81.03 ± 1.54^{a}	80.73 ± 2.78^{a}	93.70 ± 1.69^{c}	77.96 ± 1.89
ST (XII)	40.23 ± 2.05^{a}	38.53 ± 1.55^{a}	48.40 ± 2.27^{b}	44.30 ± 2.60^{ab}	56.97 ± 1.62^{c}	45.69 ± 2.02
ST (XIV)	39.67 ± 2.48^{a}	42.87 ± 1.02^{ab}	49.13 ± 2.52^{bc}	50.27 ± 1.62^{c}	56.80 ± 1.39^{d}	47.75 ± 1.81
AG (III)	71.07 ± 0.12^{a}	42.50 ± 1.85^{b}	61.30 ± 5.41^{a}	47.30 ± 5.75^{bc}	59.10 ± 2.51^{a}	56.25 ± 3.13
AG (XII)	54.97 ± 0.25^{ab}	42.93 ± 2.61^{a}	63.93 ± 5.71^{b}	48.30 ± 5.42^{a}	52.33 ± 1.95^{ab}	52.49 ± 3.19
AG (XIV)	54.87 ± 5.52^{b}	35.97 ± 3.87^{a}	52.27 ± 6.47^{b}	37.13 ± 4.03^{a}	44.63 ± 3.80^{ab}	44.97 ± 4.74
**Disulfide reductase [U cm^{-3}]**						
ST (III)	0.010 ± 0.001^{a}	0.013 ± 0.005^{a}	0.013 ± 0.005^{a}	0.019 ± 0.008^{ab}	0.032 ± 0.005^{b}	0.017 ± 0.004
ST (XII)	0.194 ± 0.008^{ab}	0.188 ± 0.012^{a}	0.223 ± 0.008^{bc}	0.207 ± 0.012^{ab}	0.256 ± 0.012^{c}	0.214 ± 0.010
ST (XIV)	0.013 ± 0.005^{a}	0.036 ± 0.009^{b}	0.019 ± 0.008^{ab}	0.026 ± 0.005^{ab}	0.084 ± 0.005^{c}	0.036 ± 0.006
AG (III)	0.045 ± 0.005^{b}	0.010 ± 0.001^{a}	0.039 ± 0.008^{b}	0.013 ± 0.005^{a}	0.071 ±0.012^{c}	0.036 ± 0.006
AG (XII)	0.013 ± 0.005^{a}	0.010 ± 0.001^{a}	0.023 ± 0.005^{ab}	0.036 ± 0.009^{b}	0.013 ±0.005^{a}	0.019 ± 0.005
AG (XIV)	0.036 ± 0.005^{b}	0.026 ± 0.005^{ab}	0.013 ± 0.005^{a}	0.071 ± 0.012^{c}	0.023 ±0.005^{ab}	0.034 ± 0.006
**Proteins and peptides [μg of proteins cm^{-3}]**						
ST (III)	297.42 ± 5.44^{b}	473.18 ± 7.52^{a}	473.37 ± 6.33^{a}	509.37 ± 7.35^{c}	545.95 ± 19.68^{d}	459.86 ± 9.26
ST (XII)	341.12 ± 20.62^{b}	421.78 ± 14.01^{a}	387.32 ± 9.27^{ab}	394.06 ± 14.68^{a}	430.25 ± 15.16^{a}	394.91 ± 14.75
ST (XIV)	308.40 ± 7.41^{d}	394.06 ± 1.91^{bc}	365.19 ± 2.37^{a}	378.85 ± 6.48^{ab}	413.12 ± 10.90^{c}	371.92 ± 5.81
AG (III)	471.45 ± 43.81^{a}	459.51 ± 10.46^{a}	529.59 ± 25.18^{ab}	571.94 ± 28.75^{bc}	630.65 ± 11.12^{c}	532.63 ± 23.86
AG (XII)	500.71 ± 18.91^{ab}	460.48 ± 21.72^{a}	495.90 ± 20.80^{ab}	517.27 ± 24.28^{ab}	529.97 ± 6.74^{b}	500.86 ± 18.49
AG (XIV)	380.97 ± 26.27^{a}	355.56 ± 3.48^{a}	398.10 ± 44.10^{a}	414.47 ± 36.13^{a}	429.87 ± 11.37^{a}	395.79 ± 24.27
**Amino groups [μg N-NH$_2$ cm^{-3}]**						
ST (III)	5.78 ± 0.43^{a}	10.62 ± 0.23^{b}	10.34 ± 0.30^{b}	6.16 ± 0.33^{a}	6.61 ± 0.19^{a}	7.90 ± 0.30
ST (XII)	7.02 ± 0.16^{b}	10.89 ± 0.15^{c}	11.98 ± 0.31^{a}	12.57 ± 0.34^{a}	14.46 ± 0.33^{d}	11.38 ± 0.26
ST (XIV)	5.37 ± 0.31^{b}	8.32 ± 0.24^{a}	9.15 ± 0.28^{a}	7.32 ± 0.17^{c}	8.42 ± 0.32^{a}	7.72 ± 0.26
AG (III)	3.63 ± 0.17^{b}	12.17 ± 1.04^{c}	17.28 ± 1.79^{d}	23.25 ± 0.73^{a}	24.20 ± 1.41^{a}	16.11 ± 1.03
AG (XII)	6.19 ± 0.22^{c}	20.81 ± 0.44^{ab}	19.56 ± 0.87^{a}	21.48 ± 1.04^{ab}	22.83 ± 1.11^{b}	18.17 ± 0.74
AG (XIV)	5.87 ± 0.50^{c}	17.60 ± 0.68^{a}	16.89 ± 0.40^{a}	20.31 ± 0.49^{b}	21.49 ± 0.39^{b}	16.43 ± 0.49
**Thiol groups [μg -SH cm^{-3}]**						
ST (III)	2.35 ± 0.15^{a}	2.99 ± 0.15^{ab}	6.72 ± 0.47^{d}	4.69 ± 0.15^{c}	3.95 ± 0.53^{bc}	4.14 ± 0.29
ST (XII)	5.28 ± 0.13^{ab}	7.14 ± 0.87^{c}	3.73 ± 0.40^{a}	4.37 ± 0.33^{a}	6.13 ± 0.42^{bc}	5.33 ± 0.43
ST (XIV)	4.80 ± 0.23^{a}	5.33 ± 0.33^{a}	4.53 ± 0.87^{a}	4.69 ± 0.53^{a}	4.74 ± 0.96^{a}	4.82 ± 0.58
AG (III)	4.90 ± 0.96^{a}	6.66 ± 0.62^{a}	6.98 ± 1.18^{a}	4.32 ± 0.69^{a}	1.07 ± 0.33^{b}	4.79 ± 0.76
AG (XII)	5.06 ± 0.67^{ab}	6.18 ± 0.20^{a}	5.60 ± 1.33^{ab}	2.88 ± 0.79^{b}	6.08 ± 0.78^{a}	5.16 ± 0.75
AG (XIV)	2.56 ± 0.35^{ab}	3.63 ± 0.20^{bc}	5.54 ± 1.63^{c}	0.69 ± 0.08^{a}	3.09 ± 0.49^{abc}	3.10 ± 0.55
**Sulfate ions [mg SO_4^{2-} cm^{-3}]**						
ST (III)	0.32 ± 0.03^{a}	0.95 ± 0.02^{d}	1.15 ± 0.02^{e}	0.55 ± 0.03^{b}	0.66 ± 0.03^{c}	0.73 ± 0.02
ST (XII)	0.59 ± 0.04^{a}	0.96 ± 0.04^{c}	0.57 ± 0.03^{a}	0.17 ± 0.01^{b}	0.53 ± 0.02^{a}	0.56 ± 0.03
ST (XIV)	0.34 ± 0.03^{a}	1.00 ± 0.06^{b}	0.91 ± 0.01^{b}	0.31 ± 0.10^{a}	0.60 ± 0.02^{c}	0.63 ± 0.04
AG (III)	0.51 ± 0.04^{b}	0.65 ± 0.03^{c}	0.96 ± 0.03^{d}	1.16 ± 0.02^{a}	1.09 ± 0.04^{a}	0.87 ± 0.03
AG (XII)	0.58 ± 0.03^{b}	0.70 ± 0.04^{b}	1.10 ± 0.04^{a}	1.10 ± 0.03^{a}	1.10 ± 0.04^{a}	0.92 ± 0.04
AG (XIV)	0.54 ± 0.03^{c}	0.64 ± 0.02^{d}	0.89 ± 0.03^{a}	0.95 ± 0.02^{ab}	0.98 ± 0.03^{b}	0.80 ± 0.03
**Ammonium ions [μg NH^{4+} cm^{-3}]**						

ST (III)	365.53 ± 7.71[c]	637.65 ± 9.38[a]	620.27 ± 23.04[a]	558.61 ± 7.19[b]	598.95 ± 12.50[ab]	556.20 ± 11.96
ST (XII)	525.24 ± 7.75[b]	687.03 ± 8.92[a]	677.99 ± 5.42[a]	555.37 ± 9.20[c]	593.61 ± 10.50[d]	607.85 ± 8.36
ST (XIV)	460.33 ± 14.23[b]	646.23 ± 2.29[a]	668.71 ± 11.49[a]	531.49 ± 8.98[c]	578.08 ± 6.02[d]	576.97 ± 8.60
AG (III)	217.19 ± 18.78[b]	256.59 ± 29.81[b]	423.25 ± 39.92[a]	433.68 ± 42.91[a]	457.32 ± 46.90[a]	357.61 ± 35.66
AG (XII)	319.17 ± 29.34[b]	411.43 ± 10.73[a]	454.31 ± 11.27[a]	417.92 ± 13.35[a]	392.42 ± 37.65[a]	399.05 ± 20.47
AG (XIV)	276.06 ± 24.59[b]	427.88 ± 10.23[a]	434.37 ± 22.21[a]	403.31 ± 6.40[a]	392.19 ± 10.33[a]	386.76 ± 14.75

Explanations: as in Table S1.
